# Harvest models of small populations of a large carnivore using Bayesian forecasting

**DOI:** 10.1002/eap.2063

**Published:** 2020-01-28

**Authors:** Henrik Andrén, N. Thompson Hobbs, Malin Aronsson, Henrik Brøseth, Guillaume Chapron, John D. C. Linnell, John Odden, Jens Persson, Erlend B. Nilsen

**Affiliations:** ^1^ Grimsö Wildlife Research Station Department of Ecology Swedish University of Agricultural Sciences SE‐730 91 Riddarhyttan Sweden; ^2^ Natural Resource Ecology Laboratory Department of Ecosystem Science and Sustainability, and Graduate Degree Program in Ecology Colorado State University Fort Collins Colorado 80523 USA; ^3^ Department of Zoology Stockholm University SE‐106 91 Stockholm Sweden; ^4^ Rovdata, Norwegian Institute for Nature Research P.O. Box 5685, Torgard NO‐7485 Trondheim Norway; ^5^ Norwegian Institute for Nature Research P.O. Box 5685, Torgard NO‐7485 Trondheim Norway

**Keywords:** adaptive management, Bayesian state‐space model, carnivore, Eurasian lynx, forecasting, harvest, hunting, Norway, poaching, quota, Sweden

## Abstract

Harvesting large carnivores can be a management tool for meeting politically set goals for their desired abundance. However, harvesting carnivores creates its own set of conflicts in both society and among conservation professionals, where one consequence is a need to demonstrate that management is sustainable, evidence‐based, and guided by science. Furthermore, because large carnivores often also have high degrees of legal protection, harvest quotas have to be carefully justified and constantly adjusted to avoid damaging their conservation status. We developed a Bayesian state‐space model to support adaptive management of Eurasian lynx harvesting in Scandinavia. The model uses data from the annual monitoring of lynx abundance and results from long‐term field research on lynx biology, which has provided detailed estimates of key demographic parameters. We used the model to predict the probability that the forecasted population size will be below or above the management objectives when subjected to different harvest quotas. The model presented here informs decision makers about the policy risks of alternative harvest levels. Earlier versions of the model have been available for wildlife managers in both Sweden and Norway to guide lynx harvest quotas and the model predictions showed good agreement with observations. We combined monitoring data with data on vital rates and were able to estimate unobserved additional mortality rates, which are most probably due to poaching. In both countries, the past quota setting strategy suggests that there has been a de facto threshold strategy with increasing proportion, which means that there is no harvest below a certain population size, but above this threshold there is an increasing proportion of the population harvested as the population size increases. The annual assessment of the monitoring results, the use of forecasting models, and a threshold harvest approach to quota setting will all reduce the risk of lynx population sizes moving outside the desired goals. The approach we illustrate could be adapted to other populations of mammals worldwide.

## Introduction

Some species of large carnivores have naturally recolonized or have been successfully reintroduced to parts of their historic range after being historically extirpated by humans (Breitenmoser et al. 2001, Smith et al. 2003, Chapron 2014). Large carnivores are conflict‐prone species and their recovery can become controversial (Carter and Linnell 2016, Chapron and Lopez‐Bao 2016). Some governments attempt to navigate the political and social challenges accompanying large carnivore recovery by enacting population goals or population targets. These often take the form of absolute maximum population numbers or narrow population intervals within which large carnivore populations should remain, as implemented in Scandinavia (Norwegian Ministry of the Environment 2003 and Swedish Ministry of the Environment 2012). In such cases, legal control and harvest can be a management tool to maintain a population within these limits. However, because large carnivores also have high degrees of legal protection, harvest quotas have to be carefully justified and constantly adjusted to avoid damaging their conservation status. Furthermore, hunting predators creates its own set of conflicts in both society and among conservation professionals (Ericsson et al. 2004, Treves 2009, Linnell et al. 2017, Macdonald et al. 2016, Lute et al. 2018), where one consequence is a need to demonstrate that management is sustainable, evidence‐based, and guided by science. In addition, because the abundance of recently recovered large carnivores is often low relative to many other wildlife species, decisions on harvest need to be made particularly carefully to prevent unwanted declines in carnivore abundance (Creel et al. 2015). Thus, it is critical to monitor carnivore populations closely and adjust harvest rates if the population falls below, or exceeds, politically determined limits for its abundance.

The choice of harvest strategies will influence the probabilities for meeting politically set goals. A threshold quota setting with increasing proportional quotas above the threshold reduces population variability and is less likely to cause unexpected declines in population size than constant harvest or strict proportional harvest, especially under uncertainty (Lande et al. 2003, Fryxell et al. 2010, Sæther et al. 2010). A threshold harvest with increasing proportion means that there is no harvest below a certain population size, but above this threshold the proportion of the population harvested increases with the population size. A strict proportional harvest means that a constant proportion of the population is harvested every year irrespective of population size.

Adaptive management (Walters 1986) provides a conceptual framework that is particularly well suited to manage small populations (Shea et al. 1998). Once management goals for abundance have been chosen, a harvest quota can be set with the aim to reach the goals; the population is monitored repeatedly; and harvest levels are adjusted if the population size exceeds or falls below the predetermined management goals. Goals for abundance of carnivore populations can be specified as a range, with the lower limit of the range set by the objective to maintain viable predator populations and the upper limit politically determined by governments.

Bayesian forecasting models that combine monitoring data with studies of vital rates of populations are particularly useful tools for informing decision makers about the ability of current management actions to meet goals for the future (Hobbs et al. 2015, Raiho et al. 2015, Ketz et al. 2016). For example, these models can inform decisions on harvest alternatives by forecasting the effect of current practices on future population sizes. In particular, these models can specify the probability that different levels of harvest will result in future populations that are below, within, or above an acceptable range of abundances. These probabilities reflect multiple sources of uncertainty arising from sampling variance and process variance.

As a supplement to the Bayesian forecasting models, which we developed and applied here, Management System Evaluation (MSE) models could be used to simulate socioecological systems wherein different management scenarios are explored (Milner‐Gulland et al. 2010, Bunnefeld et al. 2011). Such models could be particularly useful when the intention is to explore a wide set of management options, including changes to the general policy settings. This is beyond the scope of the current study, but we note that the population model developed here could be integrated into such a simulation tool, in which a broader set of policies, e.g., including changing management goals, long term changes in large carnivore population dynamics due to climate change, and various levels of illegal hunting as a response to current management regimes, are explored.

Here, we illustrate the use of a Bayesian state‐space model to guide decisions on harvesting small populations of carnivores using Eurasian lynx (*Lynx lynx*; hereafter, called “lynx”) in Sweden and Norway as an illustrative example. This is a real case example, as results from this model or similar versions have been available for wildlife managers since 2012 in both Sweden (Andrén et al. 2010, Andrén 2017) and Norway (Nilsen et al. 2011a, Tovmo et al. 2017) to guide lynx harvest quotas. The model presented estimates the probability that the forecasted population size will be below or above the management objectives at different harvest quotas and can therefore reduce the risk of deviating from the management goals. Furthermore, by combining monitoring data on population size and harvest from Scandinavian populations with known vital rates, we investigate whether the observed vital rates together with known legal harvest can explain the observed population trends, or if additional and unquantified sources of mortality need to be included to improve the fit. These additional sources of mortality include known and cryptic poaching (Andrén et al. 2006, Liberg et al. 2012) and other mortality not recorded in radio‐telemetry‐based studies of lynx demography. We also examine the past decision‐making strategies in setting lynx harvest quotas, which have been employed by various management bodies in Sweden and Norway. We test whether the past quota setting has followed strict proportional harvest or threshold harvest with an increasing proportion.

## The Study System and Materials

**Figure 1:**
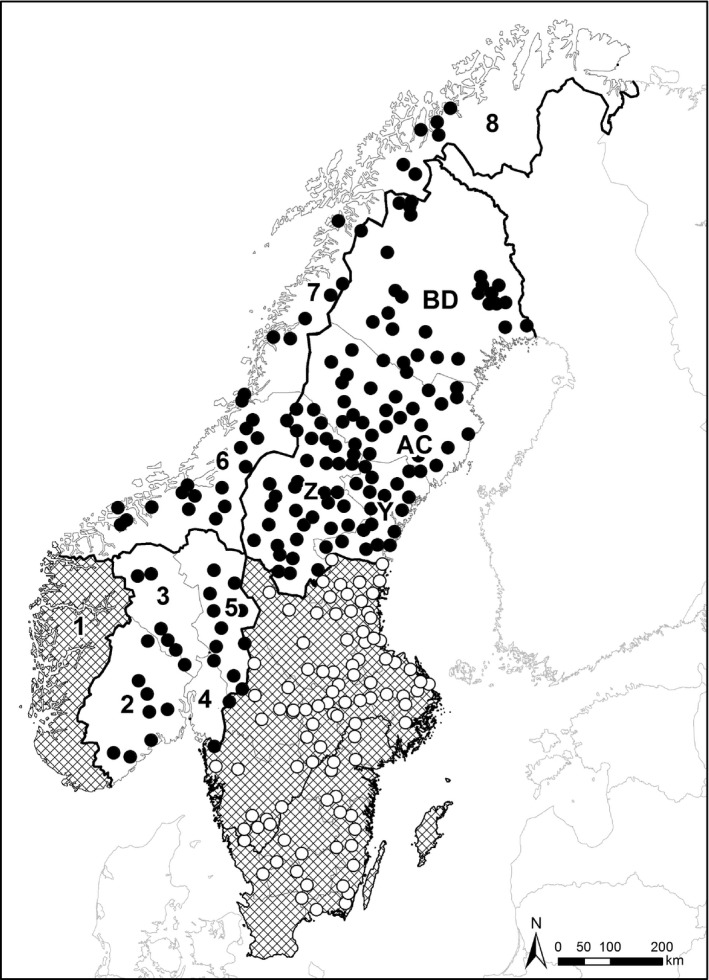
Results from monitoring of lynx family groups the winter 2016–2017 (Zetterberg and Tovmo 2017). The study area (unshaded area, black dots representing family groups) was the northern carnivore management region including the four northernmost counties (Västernorrland [Y], Jämtland [Z], Västerbotten [AC], and Norrbotten [BD]) in Sweden and the Large Carnivore Management Regions 2–8 in Norway. The area not included in this study (shaded area with open dots representing family groups) included the central and southern management regions in Sweden and management Region 1 in Norway.

The study area encompasses the northern half of Sweden and most of Norway (Fig. 1). In Sweden, the study area (225,000 km^2^) is the northern carnivore management region, which includes four counties: Västernorrland (Y), Jämtland (Z), Västerbotten (AC), and Norrbotten (BD). In Norway, the study area (273,000 km^2^) includes the large carnivore management regions 2–8 (Linnell et al. 2010). We did not include management region 1 (in the west), because the management goal is 0 lynx family groups in this management region. The southern half of Sweden was not included in our research because the lynx are more influenced by predator–prey interactions than by human harvest in this area (Andrén 2017). The study area includes alpine and boreal vegetation zones (Esseen et al. 1997), with high mountainous plateaus with peaks up to 2,500 m above sea level. Mountain birch forest (*Betula pubescens*) form the timberline at 500–900 m above sea level (higher further south). The boreal forest is dominated by conifers (Norway spruce [*Picea abies*] and Scots pine [*Pinus sylvestris*]) and most parts of the forest are intensively managed for pulp and timber, which creates a mosaic of even‐aged forest stands. The proportion of agricultural land is generally low within the study area, but increases toward the south, as does the general plant productivity.

The study area largely corresponds to the reindeer husbandry area in Sweden. In Norway, the reindeer husbandry area (140,000 km^2^) includes management regions 7 and 8 and the northern and eastern parts of management region 6 and 5 (Fig. 1). Within the reindeer husbandry area, the indigenous Sámi herd semi‐domestic reindeer (*Rangifer tarandus*). These privately owned, free‐ranging reindeer are the main prey for lynx within the reindeer husbandry area in both countries (Mattisson et al. 2011) and in northern Sweden the abundance of lynx negatively influences the reindeer harvest (Hobbs et al. 2012). Consequently, lynx predation on reindeer creates a conflict between lynx conservation and the indigenous Sámi practice of reindeer husbandry. South of the reindeer husbandry area, roe deer is the main prey for lynx (Odden et al. 2006). In Norway, lynx also prey upon free‐ranging domestic sheep, especially in the summer (Odden et al. 2008, Gervasi et al. 2014), which is the source of a major conflict. Lynx are found throughout the study area in this multi‐use, non‐wilderness landscape and coexist with many different human activities in a land‐sharing context (Phalan et al. 2011, Fischer et al. 2014).

To address the concerns among stakeholders about lynx number, both Sweden and Norway have set goals for lynx abundance through several parliamentary white papers, which have been based on consultation with different interest groups (e.g., reindeer herders, sheep farmers, hunters, and conservationists; Norwegian Ministry of the Environment 1992, 1997, 2003, Swedish Ministry of the Environment 1999, 2007, 2012). These goals are expressed as numbers of annual lynx reproductions (termed family groups) in both countries. The current national absolute minimum level for Sweden is 147 lynx family groups (corresponds to 850 individuals), which Swedish authorities consider fulfills the requirements of favorable conservation status according to obligations under the European Habitats Directive 92/43/EEC (Swedish Environmental Protection Agency 2014). To reduce the risk of coming below the minimum level, there is also a higher national management goal of 221 lynx family groups. The management goal, with intervals around, is divided between each county (Appendix S1: Table S1). The County Administrative Boards can decide about lynx harvest and set quotas if the lynx population is above the lower limit of this interval, otherwise the decision about lynx harvest is made by the Swedish Environment Protection Agency. For Norway, the current national management goal from 2004 is 65 lynx family groups. At the same time, eight management regions were established, and each management region has a specified portion of the overall goal (Appendix S1: Table S1). If the annual monitoring shows that a management region is above its goal, the Regional Carnivore Management Boards can decide about lynx harvest and set the quota; otherwise the decision about lynx harvest is the responsibility of the Norwegian Environment Agency. Thus, there might be an incentive for the County Administrative Boards in Sweden and the Regional Carnivore Management Boards in Norway to keep the lynx population within the target interval so that they retain the authority to decide about lynx harvest and quotas.

Lynx harvest is managed as a de facto license hunting system in both countries, with quotas allocated to certain management regions and where recreational hunters have the opportunity to hunt until the quota is filled during a limited season. Lynx are mainly hunted in drive‐hunts with dogs, but some are also caught in box‐traps. On average, 80% of the quota is filled in Sweden and 77% in Norway. Various mechanisms are used to prevent the quota from being exceeded and female‐specific sub‐quotas are also common (Linnell et al. 2010). In addition to a hunter harvest, management authorities can authorize the legal control of specific individuals at any time for causing damages to livestock (although it is rarely done), and livestock owners are able to shoot an animal if it is caught in the act of killing livestock.

Lynx monitoring in Sweden and Norway uses a common methodology based on unreplicated counts of family groups (Knight et al. 1995, Linnell et al. 2007a, b, Gervasi et al. 2013). The monitoring is largely based on snow‐tracking and identifying lynx tracks from two or more individuals outside the mating season, which are assumed to be a family group consisting of an adult female and young of the year (Linnell et al. 2007a). Additional observations include camera‐trap images of kittens, and any kittens shot in the early part of the hunting season or killed in traffic accidents (i.e., proof of reproduction). Criteria based on home‐range sizes and movement patterns from radio‐marked female lynx are used to separate observations of different family groups, to assure that counts of family groups are distinct (Linnell et al. 2007a, Gervasi et al. 2013). Nilsen et al. (2011b) found a good fit (*r* = 0.84) between monitored population size and reconstructed population size for Norway. Thus, the lynx monitoring provides an index of all lynx in an area.

In Sweden, personnel from the County Administration Boards perform the lynx monitoring. In Norway, the National Large Predator Monitoring Program based at the Norwegian Institute for Nature Research (NINA) coordinates lynx monitoring. The monitoring is conducted from October to the end of February, but most observations are recorded during December to February. Reindeer herders, hunters, game wardens, and the public report records of lynx tracks, but all observations of lynx tracks from two or more individuals have to be verified by authorized personnel from the County Administration Boards (Sweden) or State Nature Inspectorate (Norway) before entry into the common Swedish‐Norwegian monitoring database (Rovbase; *available online*).^1^^,^ 2 The compensation system for semi‐domesticated reindeer killed by lynx in Sweden is a risk‐based system and results from the lynx monitoring is the basis for compensation (Zabel and Holm‐Müller 2008). Therefore, there is an additional strong incentive among reindeer herders to report lynx tracks to County Administration Boards personnel. The fact that lynx tracks have to be verified by authorized personnel, prevents the risk of false reporting and overestimating the numbers.

**Figure 2:**
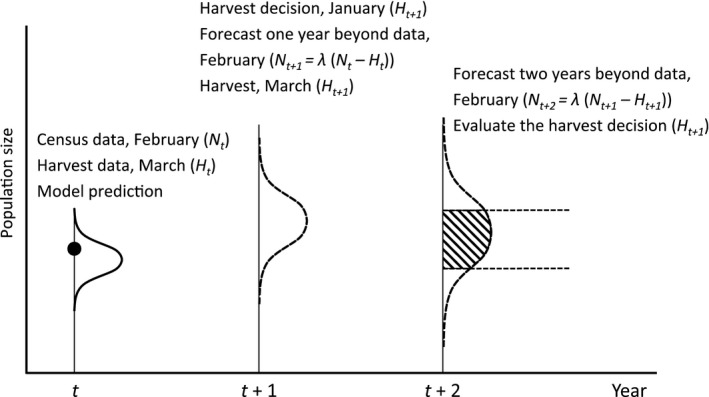
Timeline of the modeling approach used to support adaptive management of a large carnivore population. Census data (black dot, Year_*t*_ and several years before, not shown here) and prior information on carnivore vital rates are used to fit a Bayesian state‐space model. The model is used to obtain posterior distributions of the estimated population size in the past (*N*_*t*_, solid distribution curve, Year_*t*_) as well as the predictive process distribution of future populations sizes (*N*_*t+*1_ and *N*_*t+*2_, dashed distribution curve, Year_*t*+1_ and Year_t+2_). The predictive process distribution is used to forecast the effects of alternative harvest levels on the future state of the population (*N*_*t+*2_) relative to goals for abundance, shown here as an interval in population size (horizontal dotted lines). The shaded area shows the probability that a given alternative harvest level will meet goals for future abundance (*N*_*t+*2_). The harvest decision (*H*_*t+*1_) is made in January Year_*t+*1_ for the harvest that is implemented in February/March in Year_*t+*1_ and is based on the monitoring data (*N*_*t*_) from February Year_*t*_ and harvest data (*H*_*t*_) from March Year_*t*_. The effect of the harvest decision in *H*_*t+*1_ can only be evaluated after the monitoring in February *N*_*t+*2_. Thus, there is a 2‐yr lag between the input data (*N*_*t*_ and *H*_*t*_) and the possibilities to evaluate the effects of the decision (*H*_*t+*1_) on the population (*N*_*t+*2_).

An important aspect of the decision‐making process concerns the timeline of events (Fig. 2). Final decision about the size of the harvest quota is usually made in January (year *t* *+* 1) based on the lynx census completed in February of the previous year (year *t*), which in turn reflects the reproduction that occurred in May–June of year *t* − 1. Harvest starts in February/March (year *t* *+* 1) one or two months after the decision. Thus, managers set quotas based on the previous year population estimates of reproduction that occurred 20 months earlier (May–June of year *t* − 1). Census data from the current year are not available when quotas are set, as lynx monitoring ends 28 February and the lynx hunt starts 1 February (in Norway) or 1March (in Sweden). This also means that the effect of the lynx hunt in March of year *t* *+* 1 will be evaluated in the monitoring completed in February of year *t* + 2 (i.e., 2 yr beyond data). This delay, due to the monitoring system dependence on snow conditions and harvest starting after the census, reinforces the need for population models to properly capture all uncertainty in the system.

We used data from the annual lynx monitoring in Sweden (1998–2017; Tovmo et al. 2016, Zetterberg and Tovmo 2017) and in Norway (1996–2017; Tovmo et al. 2016, Zetterberg and Tovmo 2017; Data S1). These data were grouped into three geographical areas: (1) northern Sweden (the northern carnivore management region, reindeer husbandry area), (2) southern Norway (carnivore management regions 2, 3, 4, and 5), and (3) northern Norway (carnivore management regions 6, 7, 8, reindeer husbandry area), because lynx management differs between Sweden and Norway and there are ecological differences between areas inside and outside reindeer husbandry areas.

We used data on lynx shot during the annual quota hunt in Sweden and Norway. For Sweden, these data also include legal control of lynx issued by the county boards (7% of all shot lynx in Sweden). The harvest data were extracted from the common database (Rovbase; see footnotes 7 and 8) and the Swedish National Veterinary Institute, which includes information about sex and age class (i.e., determined by body mass and teeth development for young individuals (<1 yr old) and incremental lines in the tooth cementum for older individuals). We also used data on the set quotas provided by the Swedish Environmental Protection Agency and the Norwegian Environment Agency (Data S2).

We used results from radio‐telemetry studies on lynx demography in Sweden and Norway (Andrén et al. 2002, 2006, Nilsen et al. 2012, Basille et al. 2013, Gaillard et al. 2014, Walton et al. 2017) to get the vital rates for the population model. We used the survival rate from Andrén et al. (2006) not including harvest mortality, because harvest off‐take is modeled as a separate process in our model. Thus, this approach estimates the potential population growth rate without the effects of harvest.

## Models

### Deterministic lynx harvest model

We represented estimated, unobserved lynx populations in the state vector *n*_*i,j,k,t*_ using a female‐only model with three age classes. The proportion of females in the lynx population is around 0.52 ± 0.05 (mean ± SD; recalculated from Andrén et al. 2002). On average, one lynx family group represents 0.31 ± 0.023 of the total number of females in the population (recalculated from Andrén et al. 2002, *f*_*k,t*_). Subscripts *i* are used to index age class, *j* index management region, and *t* index year. The vector *n*_1*,j,k,t*_ portrays the true, unobserved number of female kittens aged 9 months at time of census, in management region *j* and at time *t*,* n*_2*,j,k,t*_ the true, unobserved number of subadult females aged 21 month at census, and *n*_3*,j,k,t*_, the true, unobserved number of adult females, aged 33 months and older at census. The *j* management regions were further grouped into *k* geographical areas (Appendix S1: Table S1), within which demographic rates in management region *j* were assumed to be from a common distribution in geographical area *k*. Births occurs as a pulse in June and the population census ends nine months later in February, and the legal quota harvest occurs in March, i.e., immediately after the census. The pre‐breeding projection matrix (end of February) for this system is

(1) $$A_{k}=\left(\begin{array}{ccc} 0 & r_{1,k}\left(\phi_{2,k}^{1/4}-\rho_{k}\phi_{2,k}^{1/8}\right) & r_{2,k}\left(\phi_{2,k}^{1/4}-\rho_{k}\phi_{2,k}^{1/8}\right)\\ \phi_{1,k}-\rho_{k}\phi_{1,k}^{1/2} & 0 & 0\\ 0 & \phi_{2,k}-\rho_{k}\phi_{2,k}^{1/2} & \phi_{2,k}-\rho_{k}\phi_{2,k}^{1/2} \end{array}\right)$$

where *r*_1_ and *r*_2_ is the number of female kittens that survive to their first census at time *t* per female 2 yr old and per female 3‐yr‐old and older alive during the breeding season at time *t* − 1; Φ_1_ is the probability of survival from age 9 months to 21 months, and Φ_2_ is the annual probability of survival of animals aged 21 months and older. We assumed non‐harvest mortality risk is constant throughout the year, an assumption supported by nonsignificant time dependence in analyzing lynx survival using a Cox proportional hazard model (*P* > 0.48; H. Andrén, *unpublished data*), so that the probability of surviving one month is $\Phi_{i}^{1/12}$. Because the surveys occur in February and because females must survive from census to the birth pulse in spring (i.e., one‐quarter of a year) in order to reproduce, the product $\Phi_{2}^{1/4}$ is included in the recruitment expression (Eq. (1)). Harvest takes place as a pulse in March removing a known number of individuals (with known sex and age categories), and therefore mortalities due to legal quota harvest are not included in the survival estimates used here (instead they are removed as a vector (*h*_*i,j,k,t*_)). The parameter ρ_*k*_ is an additional source of mortality in yearlings and adults in geographical area *k*, which could result from regional differences in poaching (Andrén et al. 2006) or other mortality not recorded in radio‐telemetry studies on lynx demography. This additional mortality could occur any time during the year and therefore multiplied by survival $\Phi_{i}^{1/2}$ *,* i.e., the additional mortality occurs on average after one‐half of a year and by survival $\Phi_{i}^{1/8}$ for the period after the survey (i.e., one‐quarter of a year). The *j* management regions were grouped into *k* geographical areas, with geographical area specific recruitment (*r*_1,*k*_ and *r*_2*,k*_) and survival (Φ_1,*k*_ and Φ_2,*k*_). A life cycle diagram for the projection matrix is shown in Appendix S1: Fig. S1. The median of the marginal posterior distribution of the population size was modeled as

(2) $$\mu_{i,j,k,t}=A_{k}\left(n_{i,j,k,t-1}-h_{i,j,k,t-1}\right)$$

where *h*_*i,j,k,t*−1_ is a vector of observed harvest of kittens and females in management region *j* immediately following time *t* − 1. The legal harvest follows immediately after the census and is therefore subtracted from the population size before multiplying the population vector with the transition matrix to get the population size in year *t*.

Eq. (2) is the simplest possible model that allowed us to exploit highly accurate data on sex and age of female harvest. A simple model is necessary for populations like lynx for which data are scarce. Models of high dimension increase the risk of overfitting and problems with identifying parameters. For example, we attempted to fit models including covariance in vital rates of age classes but these failed to converge, indicating that there was not sufficient information in the data to separately identify standard deviations for each age class and their correlations.

### Stochastic lynx harvest model

The deterministic model presented above (μ_*i,j,k,t*_) does not include many influences (e.g., density dependence, weather, disease, immigration, and emigration) that potentially affect the dynamics of wild populations. We choose to represent stochasticity using the parameter $\sigma_{p}^{2}$, which accounts for the variance in true state not accounted for by the deterministic model

(3) $$\log\left(n_{i,j,k,t}\right)∼\text{multivariate normal}\;\left(\log\left(\mu_{i,j,k,t}\right),\sigma_{p}^{2}I\right)$$

where **I** is the identity matrix. Thus, $\sigma_{p}^{2}$ **I** is a variance–covariance matrix with $\sigma_{p}^{2}$ on the diagonal and 0s elsewhere, which means that the process error is the same for all areas.

We accounted for sampling variation by relating the state vector to observations of the number of lynx family groups using

(4) $$y_{j,k,t}∼\text{Poisson}\left(f_{k,t}\times \sum_{i=1}^{3}n_{i,j,k,t}\right)$$

where *y*_*j,k,t*_ is the number of family groups in management region *j* at time *t*. The number of lynx family groups per lynx female in the population ( *f*_*k,t*_) in year *t* in geographical area *k* was drawn from a beta distribution with a mean and standard deviation of *F*_*k*_ and σ_*Fk*_, which correspond to *f*_*k,t*_ being a yearly random factor from the hyperparameter *F*_*k*_ and allows handling temporal variation to some extent.

We used results from radio‐telemetry studies on lynx demography in Sweden and Norway (Andrén et al. 2002, 2006, Nilsen et al. 2012) to extract informative prior distributions for the parameters survival without harvest mortality (**ϕ**), recruitment (**r**), mean and standard deviation of number of family groups per total female lynx population (**F** and **σ**_**F**_), and initial age composition (ψ) in the state and observation equations (Eqs. (1) and (4); Table 1). Vague prior distributions were assigned to additional mortality (**ρ**) and process error (σ_*p*_; Table 1).

**Table 1:** Prior distributions of demographic parameters in the lynx model; harvest mortality is not included in the survival estimates

Parameter	Definition	Distribution	Mean	SD	Source
Φ_1_	probability of survival of subadult females	beta (9.1, 1.01)	0.90	0.090	Andrén et al. (2006)
Φ_2_	probability of survival of adult females	beta (20.5, 0.74)	0.96	0.039	Andrén et al. (2006)
*r*_1,1&3_	number of female kittens surviving to census per 2‐yr‐old female geographical areas 1 and 3	beta (4, 14)	0.19	0.098	Nilsen et al. (2012); updated H. Andrén and J. Odden, *unpublished data*
*r*_2,1&3_	number of female kittens surviving to census per 3‐yr‐old and older female geographical areas 1 and 3	beta (55, 85)	0.39	0.041	Nilsen et al. (2012); updated H. Andrén and J. Odden, *unpublished data*
*r*_1,2_	number of female kittens surviving to census per 2‐yr‐old female geographicalarea 2	beta (9, 9)	0.50	0.12	Nilsen et al. (2012); updated J. Odden, *unpublished data*
*r*_2,2_	number of female kittens surviving to census per 3‐yr‐old and older female geographical area 2	beta (53, 53)	0.50	0.049	Nilsen et al. (2012); updated J. Odden, *unpublished data*
ρ_*k*_	additional mortality in geographical area *k*	uniform (0, 1)			
*F*_*k*_	mean number of family groups per total number of females in population in geographical area *k*	beta (126, 278)	0.31	0.023	calculated from Andrén et al. (2002)
σ_*Fk*_	standard deviation of number of family groups per total number of females in population in geographical area *k*	beta (20.7, 877)	0.023	0.005	calculated from Andrén et al. (2002)
ψ	age composition of females for initial conditions	Dirichlet(3.15, 2.48, 9.16)			
σ_*p*_	process standard deviation on log scale	uniform (0, 4)			

Notes

Geographical area (*k*): northern Sweden is coded as 1, southern Norway (Management regions 2–5) as 2, and northern Norway (Management regions 6–8) as 3.

Whenever possible, it is preferable to use priors that are informative in Bayesian analyses (Hobbs and Hooten 2015). We evaluate the sensitivity of model results to priors in two ways. We overlay plots of posteriors on priors to reveal the influence of priors on posteriors, a standard, best practice reporting in Bayesian analysis (Hobbs and Hooten 2015). Strong overlap between prior and posterior indicates that the new data failed to change knowledge of the parameter. However, even when new data do not meaningfully change the posterior relative to the prior, the prior can allow the data to inform other parameters, latent states, and derived quantities that would otherwise be inestimable (Hobbs et al. 2015).

We also included an evaluation of sensitivities to priors by using vague priors (uniform(0,1)) for one demographic parameter at a time and observed the effect on potential growth rate (λ) compared with the original population model with informed priors (Appendix S1: Table S2). The sensitivity of the number of family groups per total female lynx population was tested by increasing the standard deviation by 30%. We also tested the sensitivity in initial conditions by increasing and decreasing the initial census results by 30% (Appendix S1: Table S2).

Initial conditions were estimated using the observed number of family groups in the first year multiplied by the stable age‐distribution of the lynx population obtained from the dominant right eigenvector of a transition matrix parameterized using the mean survival and recruitment rates shown in Table 2. We then modeled

(5) $$\begin{array}{cc} \psi & ∼\text{Dirichlet}(3.15,2.48,9.16)\\ n_{i,j,k,1} & ∼\psi y_{i,k,1}/f_{k,1} \end{array}$$

making ψ vague by assuming an initial sample size of 10.

**Table 2:** Statistics summarizing posterior distributions of demographic parameters in the lynx model (Eq. (6)), with a 95% equal‐tailed Bayesian credible interval (BCI)

Parameter	Definition	Geographical area†	Mean (±SD)	95% BCI	Median
Φ_1_	survival of subadult females		0.89 (±0.092)	0.66–0.99	0.91
Φ_2_	survival of adult females		0.98 (±0.026)	0.91–0.99	0.98
*r*_1,1_	number of female kittens surviving to census per 2‐yr‐old female	1	0.24 (±0.10)	0.07–0.45	0.23
*r*_2,1_	number of female kittens surviving to census per 3‐yr‐old and older female	1	0.40 (±0.041)	0.32–0.48	0.39
*r*_1,2_	number of female kittens surviving to census per 2‐yr‐old female	2	0.46 (±0.11)	0.25–0.69	0.46
*r*_2,2_	number of female kittens surviving to census per 3‐yr‐old and older female	2	0.49 (±0.048)	0.40–0.59	0.49
*r*_1,3_	number of female kittens surviving to census per 2‐yr‐old female	3	0.24 (±0.10)	0.08–0.46	0.23
*r*_2,3_	number of female kittens surviving to census per 3‐yr‐old and older female	3	0.40 (±0.040)	0.32–0.48	0.40
ρ_1_	additional mortality	1	0.18 (±0.032)	0.11–0.23	0.18
ρ_2_	additional mortality	2	0.088 (±0.036)	0.02–0.16	0.088
ρ_3_	additional mortality	3	0.058 (±0.033)	0.01–0.13	0.056
*F*_1_	number of family groups per total number of females in the population	1	0.31 (±0.022)	0.27–0.36	0.31
*F*_2_	number of family groups per total number of females in the population	2	0.29 (±0.023)	0.24–0.33	0.29
*F*_3_	number of family groups per total number of females in the population	3	0.30 (±0.021)	0.26–0.34	0.30
σ_*p*_	process standard deviation on log scale		0.25 (±0.030)	0.19–0.31	0.25
λ_1_	potential population growth rate	1	1.01 (±0.023)	0.96–1.05	1.01
λ_2_	potential population growth rate	2	1.19 (±0.030)	1.13–1.25	1.19
λ_3_	potential population growth rate	3	1.16 (±0.032)	1.10–1.23	1.16

Notes

Harvest mortality is not included in the survival estimates.

†Geographical area: northern Sweden is coded as 1, southern Norway (Management regions 2–5) as 2, and northern Norway (Management regions 6–8) as 3.

We approximated the marginal posterior distributions of latent states and parameters using

(6) $$\begin{array}{cc} & \left[\phi ,r,F,\rho ,n,\sigma_{p}^{2}|y\right]\propto \\ & \prod_{i=1}^{3}\prod_{j=1}^{J_{k}}\prod_{k=1}^{3}\prod_{t=2}^{T_{j,k}}\left[\log\left(n_{i,j,k,t}\right)|\log\left(A_{k}\left(n_{i,j,k,t-1}-h_{i,j,k,t-1}\right)\right),\sigma_{p}^{2}I\right]\\ & \times \prod_{t=1}^{T_{j,k}}\left[y_{j,k,t}|\;f_{k,t}\times \sum_{i=1}^{3}n_{i,\;j,k,t}\right]\left[f_{k,t}|F_{k}\right]\left[\phi \right]\left[r\right]\left[F\right]\left[\rho \right]\left[\sigma_{p}^{2}\right]\\ & \times \left[n_{1,\;j,k,1}\right]\left[n_{2,\;j,k,1}\right]\left[n_{3,\;j,k,1}\right] \end{array}.$$

### Forecasting

Bayesian state‐space models are particularly well suited to forecasting the behavior of dynamic and uncertain systems. We use the term forecast to mean predictions of the future state of a system accompanied by rigorous estimates of uncertainty (Dietze 2017). Given a sufficient time series of observations, Bayesian methods allow us to approximate posterior distributions of parameters in a model of processes controlling population abundance, and the posterior distributions of the true, unobserved state of the population in the past. We can then use the process model to make forecasts of the future state of the population if the uncertainty in observations can be properly separated from uncertainty in our model of the population processes. We make inference on future states using predictive process distributions (Hobbs and Hooten 2015), described in greater detail in the following subsection.

Recent work on large mammals (Hobbs et al. 2015, Raiho et al. 2015, Ketz et al. 2016) used predictive process distributions to evaluate the probability that thresholds for population states would be surpassed at different times in the future given different management actions, an approach with precedent in fisheries stock assessments (McAllister et al. 1994, Meyer and Millar 1999a, b, 2000a, 2000b, Millar 2011). Predictive process distributions can inform decisions on harvest alternatives by forecasting their effects on future population sizes and by computing the probability that different levels of harvest will result in future populations that are below, within, or above an acceptable range of abundances. Predictive process distributions have recently been applied to guide harvest of abundant mammals (Raiho et al. 2015, Nowak et al. 2018), but here we apply this approach to inform decisions on harvesting rare species. This is an important application because governments consider that regulating the abundance of such species may be necessary to prevent conflict with some stakeholders, but harvest must be carefully managed to assure that goals for recovering species are met. A prudent approach to harvesting small populations requires us to properly quantify the risk associated with harvest so that managers can then implement political decisions without exposing populations to high risk. An adaptive approach based on short‐term forecasts offers a promising approach to this dilemma by quantifying the risk associated with different levels of harvest. This application differs from managing sustained yield of abundant, commercial resources like fisheries, as it is applied to a conservation context.

Predictive process distributions are marginal distributions of future states of the population (Hobbs and Hooten 2015) providing forecasts with accompanying estimates of uncertainty. We defined *T* as the final year with data. For notational convenience, we defined **θ** as a vector of model parameters, **θ = (Φ**,***r***,** ρ**, $\sigma_{p}^{2}$)′ and *m* as the length of **θ**. The predictive process distribution of the elements of the state vector **n** for a single population at time *T* + 1 is

(7) $$\begin{array}{cc} & [n_{j,k,T+1}|y_{j,k}]=\int \int \ldots \int \left[n_{j,k,T+1}|n_{j,k,T},\theta \right]\\ & \left[n_{j,k,1},\ldots ,n_{j,k,T},\theta_{1},\ldots ,\theta_{m}|y_{j,k,1},\ldots {,y}_{j,k,T}\right]\\ & \times d\theta_{1},\ldots ,d\theta_{m},dn_{j,k,1},\ldots ,dn_{j,k,T} \end{array}$$

which computationally requires sampling $n_{j,k,T}^{\left(\kappa \right)}$ from [**n**_*j*,*k*,*T*+1_|$n_{j,k,T}^{\left(\kappa \right)}$, **θ** ^(κ)^] at each Monte Carlo Markov Chain (MCMC) iteration κ. The predictive process distribution for the total population size is computed as a derived quantity $\Sigma_{i=1}^{3}$ $n_{i,j,k,T}^{\left(\kappa \right)}$. The probability of meeting goals for a specified range of population sizes is found empirically as the proportion of the converged MCMC iterations for which $\Sigma_{i=1}^{3}$ $n_{i,j,k,T}^{\left(\kappa \right)}$ falls within the desired range (Fig. 2).

### Forecasting lynx populations with different harvest levels

In order to illustrate the utility of our model, we forecasted the lynx population 1 yr beyond the last data point that we had available (to February 2018) using data on the number of family groups in the last census (February 2017) and the harvest just after the last census (February/March 2017). We then forecasted the lynx population 2 yr beyond the data (to February 2019) at different harvest levels (in February/March 2018; Fig. 2). We assumed four different harvest levels (0, 5, 10, and 20 lynx) for each management region. The harvest quotas were not divided into sex and age classes; instead the age‐ and sex composition of the harvest was based on the observed composition of the harvest data. The lynx harvest is male biased and among shot lynx, the proportion of males was 0.57 ± 0.20, the proportion of females older than 21 months was 0.34 ± 0.19 and the proportion of female kittens was 0.09 ± 0.11. To model the effect of harvest we randomly assigned harvest into males and females based on their proportions in the harvest data. The proportions of female kittens (9 months old) and females older than 21 months in the harvest bag were based on the harvest data and the further division of the proportion of females aged 21 months and females aged 33 months and older was based on their estimated proportion in the modeled population matrix **A** (Eq. (1)) following a Dirichlet distribution (Eq. (5)).

### Lynx quota decision model

We modeled the lynx quota in relation to the lynx census data 1 yr before to evaluate the quota setting strategies used by the managers. In a strict proportional quota setting strategy (i.e., a constant given proportion of population is harvested every year) there will be a linear relationship between quota and census data with an intercept equal to 0 (model 1; proportional quota setting strategy). A threshold‐harvest quota setting strategy with an increasing proportional harvest means that there is a threshold in population size (*X*), below which the quota will be 0 (model 2; threshold quota setting strategy). One way to describe a threshold‐harvest quota setting strategy with an increasing proportional harvest is to fit a linear relationship between population size and the set quota. This relationship will have an intercept < 0 and the slope steeper than the slope for strict proportional harvest. The threshold in population size (*X*) below which the quota will be 0, is set by 0 = *b*_0_ + *b*_1_ *X*. Solving for *X* gives the threshold value equals −*b*_0_ */b*_1_, but only for *b*_0_ *<* 0. We estimated the threshold value by calculating the ratio −*b*_0_ */b*_1_ and estimated the probability that it overlapped with 0.

We used two Bayesian models to compare these two quota‐setting strategies in Sweden and Norway, with separate models for each country. The two models are identical except that model 1 (strict proportional quota setting strategy) does not include the intercept (*b*_0_)

(8) $$\begin{array}{cc} & H_{i}∼\log\mathrm{normal}\left(\max\left(0,\log\left(b_{0}+b_{1}x_{i-1}\right),\sigma_{q}^{2}\right)\right)\\ & q_{t}∼\mathrm{Poisson}\left(H_{t}\right) \end{array}$$

where *q*_*t*_ is the set quota at time *t*,* x*_*t−*1_ is the number of family groups at time *t* − 1. The intercept is *b*_0_ (not included in model 1) and *b*_1_ is the slope. The model prediction is *H*_*t*_, $\sigma_{p}^{2}$ is the process error, and **b** is a vector of regression coefficients. Vague prior distributions were assigned to **b** *˜* normal(0, 3,000) and σ_*q*_ ~ uniform(0, 4).

### Model fitting and evaluation

We approximated the marginal posterior distributions of parameters and latent states by fitting the models to data using the Markov chain Monte Carlo algorithm implemented in JAGS using the rjags and coda package (Plummer 2003; Eqs. (6) and (8)). We ran three chains of 100,000 iterations following a 50,000 burn‐in. Convergence was checked by visual inspection of trace plots and by the diagnostics of Brooks and Gelman (1997). We used posterior predictive checks to evaluate lack‐of‐fit between models and data using Bayesian *P* values. For the lynx harvest model, evidence for temporal dependence in the residuals was evaluated using the ACF (autocorrelation functions) implemented in R (R Core Team 2018). Evidence for spatial dependence was evaluated by semi‐variograms using the spacetime package (Pebesma 2012). We approximated the posterior distribution of population growth rate (λ) by computing the dominant eigenvalue of the projection matrix **A** at each iteration of the MCMC algorithm. Note that this approach estimates the potential population growth rate and excludes the effects of harvest. Bayesian credible intervals (BCI) were computed on parameters and latent states as the interval between the 2.5% and 97.5% quantiles.

## Results

### Model evaluation

Posterior predictive checks showed that the harvest model (Eq. (6)) and the quota decision model (Eq. (8)) were able to simulate data that were consistent with the observations. For the harvest model, Bayesian *P* values for discrepancy statistics fell between 0.63 and 0.89 for all management regions except management region 3 in Norway (Bayesian *P* = 0.96), suggesting a lack of fit in management region 3. There was no evidence of temporal autocorrelation (Appendix S1: Fig. S6) or spatial autocorrelation (Appendix S1: Figs. S7 and S8) in the residuals for the harvest model. Gelman diagnostics (Brooks and Gelman 1997) of all chains had upper confidence limits on scale reduction factors <1.11.

For the quota decision model Bayesian *P* values for discrepancy statistics fell between 0.48 and 0.50. The upper confidence limits were <1.07 on scale reduction factors for all chains for Gelman diagnostics.

**Figure 3:**
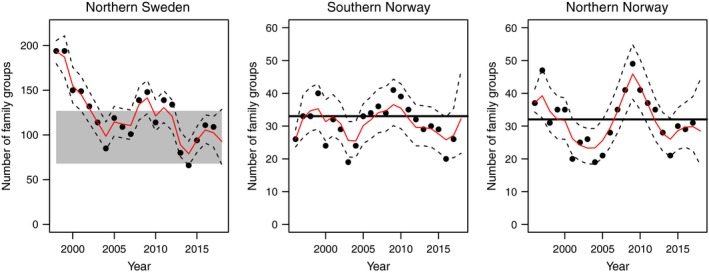
Medians of posterior distributions of the estimated number of lynx family groups in northern Sweden and southern and northern Norway (solid red line) and 95% equal‐tailed Bayesian credible intervals (dashed lines). Black dots show the monitoring data. A model forecast extends 1 yr beyond the data. Gray shaded bar shows the acceptable upper and lower limits for the number of lynx family groups in northern Sweden and the black line shows the objectives for number of lynx family groups in southern and northern Norway.

Overlays of estimates of the model fit of the estimated number of lynx family groups in Sweden and Norway showed good agreement with observations (Fig. 3). The failure of the medians of the posterior distribution to overlap all observations is a desirable outcome because it shows the model adds value to the observations by exploiting information in priors and in the full time series. The 95% Bayesian credible intervals of the number of family groups overlapped management objectives for all 11 management regions in both countries at the end of the time series (i.e., 2017; Fig. 3; Appendix S1: Fig. S2).

### Lynx model parameters

Posterior distributions of model parameters (Table 2) overlapped prior distributions in cases where priors were informative and showed minor overlap where priors were chosen to be vague (Appendix S1: Fig. S3). Decreased standard deviation of the posterior distribution relative to the prior and/or changes in its mean demonstrated that the data informed parameters beyond the information contained in the priors for the parameters (Appendix S1: Fig. S3).

An additional source of mortality in the population model was included in the population matrix to fit the observed trends in lynx abundance evident from the monitoring data. The additional mortality was highest for northern Sweden (ρ_1_; median = 0.18; Table 2). The median additional mortality in southern Norway (ρ_2_) was 0.088, whereas the median additional mortality in northern Norway (ρ_3_) was 0.056. The probability that the derived survival (Φ_*i*_ − ρ_*k*_ $\Phi_{i}^{1/2}$), which includes both the survival estimates (Φ_*i*_) and the additional mortality (ρ_*k*_), was lower in northern Sweden than in northern and southern Norway was 0.99 (Table 3). There was a 0.80 probability that the derived survival in southern Norway was lower than in northern Norway. The derived survival estimates for subadult and adult females were considerably lower than the prior survival for both of the age classes and in all three areas (Table 3). This means that monitoring data informed the survival parameters, which did not include unknown sources of mortality like poaching, beyond the information contained in the priors for survival (Appendix S1: Fig. S4).

**Table 3:** Statistics summarizing posterior distributions of the derived survival estimates (Φ_*i,k*_ − ρ_*k*_ $\Phi_{i,k}^{1/2}$ ) in the lynx model, with a 95% equal‐tailed Bayesian credible interval (BCI), the prior survival (from Table 1) and the probability that the posterior derived survival was lower than the prior survival

Female stage	Posterior derived survival mean (±SD)	Posterior derived survival 95% BCI	Prior survival mean (±SD)	*P* (posterior lower than prior survival)
Northern Sweden				
Subadult	0.73 (±0.069)	0.56–0.83	0.90 (±0.090)	0.93
Adult	0.80 (±0.028)	0.74–0.86	0.96 (±0.039)	0.99
Southern Norway				
Subadult	0.81 (±0.071)	0.65–0.92	0.90 (±0.090)	0.81
Adult	0.89 (±0.035)	0.82–0.96	0.96 (±0.039)	0.92
Northern Norway				
Subadult	0.84 (±0.078)	0.66–0.96	0.90 (±0.090)	0.73
Adult	0.92 (±0.032)	0.85–0.98	0.96 (±0.039)	0.85

*Note*

Harvest mortality was not included.

The lower derived survival rate in northern Sweden resulted in a lower potential population growth rate (λ) in northern Sweden (Table 2). There was a 0.99 probability that the potential population growth rate in northern Sweden (median = 1.01; Table 2; Appendix S1: Fig. S5), was lower than in southern Norway (median = 1.19) and northern Norway (median = 1.16). There was a 0.69 probability that the potential growth rate in northern Norway was lower than in southern Norway.

The potential growth rate (λ) was marginally affected by using vague priors for one demographic parameter at a time. The largest effect was for the parameters *r*_2_ (number of female kittens surviving to census per 3‐yr‐old and older females) where mean potential growth rate decreased and the standard deviation increased compared to the original model; from 1.19 ± 0.030 to 1.18 ± 0.032 in the alternative model in southern Norway (Appendix S1: Table S2). Neither an increased standard deviation for the parameter *F*_*k*_ (number of family groups per total number of female lynx in the population) nor a decrease or increase in the initial population size (*y*_*j,k,*1_) by 30% had any large effect on the potential growth rate (λ; Appendix S1: Table S2).

### Lynx quota setting strategy

**Figure 4:**
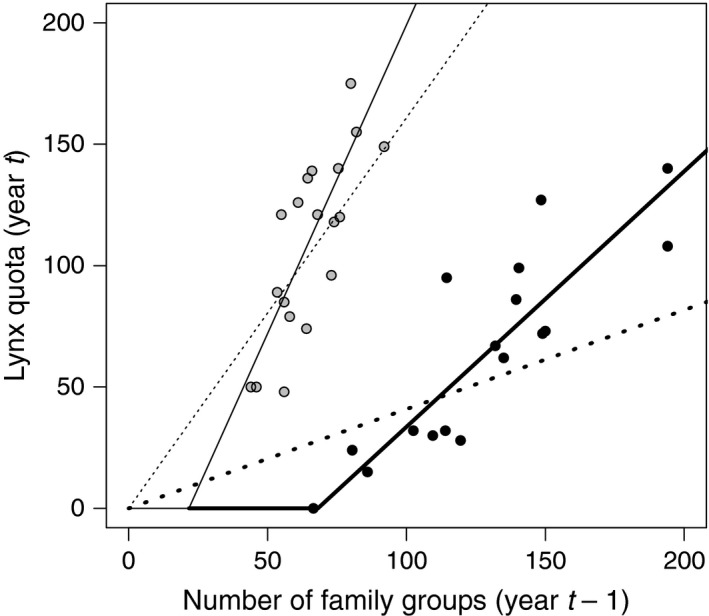
Lynx quota in year *t* in relation to census results in year *t* − 1. Black dots show observations (number of family groups) for northern Sweden and gray dots for Norway. Dashed lines indicate proportional harvest quotas (no intercept; model 1), whereas black lines indicate threshold and increasing proportional harvest quotas (model 2). Thick lines are the regression lines for northern Sweden and thin lines for Norway.

In both countries, the quotas in year *t* were positively related to the census data in year *t* − 1 (Fig. 4). The predicted quotas were higher in Norway than in northern Sweden (probability > 0.999). The slopes in model 2 (threshold quota setting strategy) were steeper than the slopes in model 1 (proportional quota setting strategy) for both northern Sweden (probability > 0.999) and Norway (probability 0.97; Table 4, Fig. 4; Fig. S9). The derived quantity (−*b*_0_ */b*_1_) for estimating the threshold in number of lynx family groups, below which there will be no harvest, based on past lynx quota decisions, was higher for northern Sweden (69 ± 2.4 SD) than for Norway (22 ± 9.3 SD; Table 4). The probability for the threshold value to be below 0 was <0.001 for northern Sweden and 0.028 for Norway. The threshold harvest with increasing proportion means that a larger proportion of the lynx population will be harvested when the lynx population increases and will cause a decline in the lynx population if the harvest is larger than the potential population growth. The threshold means that there will be no harvest below this population size and the lynx population can increase. A higher harvest quota, a lower threshold, and a slope closer to a proportional harvest in Norway compared to northern Sweden, shows that the quota setting strategy has had a larger impact on the lynx population in Norway (Fig. 4).

**Table 4:** Statistics summarizing posterior distributions of parameters in the lynx quota decision models (Eq. (7)), with a 95% equal‐tailed Bayesian credible interval (BCI)

Parameter	Mean (±SD)	95% BCI
Northern Sweden		
Model 1 (proportional harvest quota)		
*b*_1_	0.41 (±0.083)	0.27–0.60
Model 2 (threshold harvest quota)		
*b*_0_	−75.1 (±11.3)	−101.2 to −56.4
*b*_1_	1.09 (±0.14)	0.85–1.40
Threshold		
−*b*_0_ */b*_1_	69.0 (±2.4)	64.7–74.3
Norway		
Model 1 (proportional harvest quota)		
*b*_1_	1.61 (±0.10)	1.41–1.82
Model 2 (threshold harvest quota)		
*b*_0_	−59.2 (±29.2)	−115.3 to 0.33
*b*_1_	2.56 (±0.49)	1.58–3.52
Threshold		
−*b*_0_ */b*_1_	21.6 (±9.3)	−0.20 to 33.1

*Note*

The derived quantity (*−b*_0_ */b*_1_) is the estimated threshold in number of lynx family groups below which there will be no harvest based past lynx quota decisions.

### Forecasting lynx populations and effect of harvest

We forecasted the lynx population 1 yr beyond the data (to February 2018), including the known harvest during 2017. Summed over all management regions the median forecasted lynx population for northern Sweden was 93 family groups. The probabilities that the population would fall below the lower objective range in northern Sweden (68 family groups) during 2018 were 0.03 and 0.03 to be above the higher objective range (127 family groups, Table 5 and Fig. 3). The median forecasted population for southern Norway was 32 family groups. The probability that the population would be below the management objective (33 family groups) was 0.55. For northern Norway, the median forecasted population was 29 family groups and a probability of 0.70 to be below the management objective (32 family groups). The model also suggest that the forecasted lynx population in some management regions (Management regions 2, 4, 7 and 8 in Norway) will be below the management objectives in 2018 (Appendix S1: Table S3 and Appendix S1: Fig. S2).

**Table 5:** Forecasting number of lynx family groups 1 and 2 years beyond the data

Time, management region, objective†	Harvest	Median	95 % BCI	*P*		
				Below	Within	Above
1 yr beyond data (2018)						
Northern Sweden (68–127)		92	67–129	0.03	0.94	0.03
Southern Norway, regions 2–5 (33)		32	22–47	0.55		0.45
Northern Norway, regions 6–8 (32)		28	18–44	0.70		0.30
2 yr beyond data (2019)						
Northern Sweden (68–127)	0	96	65–143	0.04	0.88	0.08
	20	93	63–139	0.06	0.88	0.06
	40	91	61–136	0.08	0.87	0.05
	80	85	56–129	0.15	0.82	0.03
Southern Norway, regions 2–5 (33)	0	39	25–61	0.22		0.78
	20	36	22–57	0.35		0.65
	40	33	19–54	0.50		0.50
	80	27	14–47	0.76		0.24
Northern , regions 6–8 (32)	0	34	20–57	0.40		0.60
	15	32	18–54	0.51		0.49
	30	29	16–51	0.62		0.38
	60	25	12–46	0.79		0.21

Notes

Management objectives, the median number of lynx family groups in 2018 and 2019 with 95% equal‐tailed Bayesian credible intervals (BCI), including the known lynx harvest that occurred during 2017 for the forecast to 2018 and assuming four different harvest levels during 2018 for the forecast to 2019. *P* gives the probability that the future number of lynx family groups will be below, within, or above management objectives for a management region at the given harvest level.

Objectives appear in parentheses.

Forecasts 2 yr beyond the data (to February 2019) with different harvest levels in 2018 suggests that, summed over all counties in northern Sweden, there is a 0.04 probability that the population will be below the lower management objective with no harvest and a 0.15 probability given a harvest of 80 lynx (Appendix S1: Table S4). However, the probabilities to fall below the objective vary between management regions (Appendix S1: Table S4). For one management region (Y), the probability of falling below the objective is as high as 0.39 even with no harvest. The estimated median growth rate (λ) of 1.01 (Table 2; Appendix S1: Fig. S5) is consistent with the forecast that the lynx population is likely to remain below the objective even in the absence of harvest (Appendix S1: Table S4). For two management regions (AC and BD), there is a high probability to be within the management intervals even with some lynx harvest in 2018. In one management region (Z) there is a high probability that the population would be above the upper objective even with some harvest.

The forecast for Norway differs from the Swedish forecast, as the estimated median potential growth rate without harvest (λ) was 1.19 for southern Norway and 1.16 for northern Norway (Table 2). These positive potential growth rates suggest that the lynx population would grow rapidly if harvest were suspended. Summed over management regions in southern Norway, the probabilities that the population would be below the management objective in 2019 were 0.22 with no lynx harvest and 0.35 given a harvest of 20 lynx (Table 5). For northern Norway, the probability to fall below the management objective was 0.40 in 2019 with no lynx harvest and 0.51 given a harvest of 15 lynx (Table 5).

However, the probabilities to fall below the management objectives vary between the management regions in Norway. The number of lynx in four management regions (2, 4, 7, and 8) was below the management objective in 2017 and would most likely remain below objectives in 2018 and 2019 even with no harvest in 2018. Based on our forecast, on the one hand, there can be some (5–10 individuals) lynx harvest in three management regions (3, 5, 6) in Norway during 2018 and still result in a high probability of meeting management objectives in these management regions in 2019 (Appendix S1: Table S5). On the other hand, having no harvest in 2018 would reduce the probability that the population would be below management objectives in 2019 in these four management regions (Tables S4 and S5).

The higher probability to come below the management objective in Norway in 2019 compared to northern Sweden is a prolonged result of the previous year lynx harvest. The forecasted population for northern Sweden in 2018 is 93 (67–129, 95% BCI) lynx family groups, which is within the management interval (68–127 family groups). For Norway, the forecasted population in 2018 is 61 (46–82, 95% BCI) lynx family groups, which is lower than the management objective (65 family groups).

## Discussion

We present a Bayesian forecasting model developed to support adaptive management of large carnivores, using lynx harvesting in Scandinavia as an illustrative example. This model is based on annual monitoring data of lynx abundance (Tovmo et al. 2016, Zetterberg and Tovmo 2017) and is supported by detailed long‐term research on lynx biology, which has provided detailed estimates of key demographic parameters (Andrén et al. 2002, 2006, Nilsen et al. 2012, Basille et al. 2013, Gaillard et al. 2014, Walton et al. 2017). We used the model to estimate the probability that the forecasted population size will be below or above the management objectives when subjected to different harvest quotas. The model suggests that harvest might be possible in some regions (regions Z, AC, and BD in Sweden and regions 3, 5, and 6 in Norway) but not in others (region Y in Sweden and regions 4, 7, and 8 in Norway) to reach the management goals (Tables S4 and S5). The annual assessment of the monitoring results, updating the forecasting models and threshold harvest quota approach will all reduce the risk of inadvertently obtaining undesirable lynx population sizes in Sweden and Norway. Setting harvest quotas on a small population, which might influence its conservation status, like Eurasian lynx in Sweden and Norway, is different from managing sustainable yield of abundant and commercial resources like fisheries. Furthermore, the model we developed here can form the basis for the resource model in Management System Evaluation (MSE) models constructed to explore a wide set of management options like changes in general policy settings (Milner‐Gulland et al. 2010, Bunnefeld et al. 2011).

The essence of managing natural resources is to choose among alternative actions in terms of their ability to meet clearly specified goals. In adaptive management systems (Walters 1986), one uses monitoring data and models to constantly update these choices. Observing the responses of systems to management can improve the ability of models to predict the consequences of current actions for future system behavior and, in so doing, improve the precision of future management. Here we have illustrated how Bayesian models can be used to annually inform on harvest levels appropriate for meeting politically set goals for the desired abundance of lynx populations in Norway and Sweden.

The lynx population in northern Sweden and Norway is relatively small, with fewer than 150 family groups registered annually (corresponding to ~700–1,000 individuals), in the management regions we studied (Zetterberg and Tovmo 2017). Therefore, decisions made with respect to harvest quotas need to be made particularly carefully to prevent unwanted declines in abundance that might jeopardize the population's conservation status. In Norway there is strong evidence for positive growth rates in the lynx population in the absence of harvest, the estimated potential growth rate (*λ*) without harvest were 1.19 ± 0.030 and 1.16 ± 0.032 for southern and northern Norway. This means that the lynx population can recover rapidly if there is no harvest. On the other hand, as the estimated potential growth rate (λ) without harvest was 1.01 ± 0.023 for northern Sweden, we cannot rule out the possibility that the lynx population would be stable or declining even in the absence of harvest. Thus, the consequences of over‐harvesting lynx in northern Sweden is more serious from a conservation point of view than in Norway, as the potential growth rate (λ) without harvest was much lower in northern Sweden.

Results from the model we describe here, and earlier versions, have been available for wildlife managers since 2012 in both Sweden (Andrén et al. 2010, Andrén 2017) and Norway (Nilsen et al. 2011a, Tovmo et al. 2017) to guide lynx harvest quotas. Harvest levels in northern Sweden and Norway have been adjusted adaptively in response to monitoring results and to make forecasts of the impacts of alterative harvest quotas on lynx population sizes. The quota decision‐making is also highly relevant for lynx management, as the level of quota filling in the annual lynx harvest is high in the study area, on average 80% and 77% of the quota is filled in northern Sweden and Norway (Nilsen et al. 2011b, Bischof et al. 2012). The approach seems to be working because the Bayesian credible intervals of the number of family groups overlapped management objectives in all of the 11 management regions in both countries at the end of the time series (i.e., 2017).

Harvesting large carnivores has often been successful in limiting their abundance and distribution (Linnell et al. 2010, Swenson et al. 2017). However, the evidence that harvest per se enhances the tolerance of carnivores by people is equivocal (Treves 2009, Linnell et al. 2017). The number of lynx within a reindeer management unit in northern Sweden influenced the reindeer harvest (Hobbs et al. 2012), suggesting that a reduction in lynx numbers would increase reindeer harvest. Similarly, losses of lambs were related to lynx population size on the county level in southern Norway (Herfindal et al. 2005) and sheep losses in Norway are related to the size of the large carnivore population (Mabille et al. 2016). Harvest of carnivores is most likely to be accepted by the public if it can reduce the losses of livestock without threatening the carnivore population conservation status (Ericsson et al. 2004). Therefore, it is very important to closely monitor the lynx population and adjust harvest quotas to the monitoring results, as is done in both Sweden and Norway in an adaptive management framework. Furthermore, both Sweden and Norway have quantitative management objectives for lynx, and a management system that takes responsive actions based on the monitoring results, which will simplify management decisions (Schultz and Nie 2012, Redpath et al. 2013).

A weak connection between population size, harvest quotas and quota filling performance will lead to increased population variability. To reduce the risk of undesirable population variability as well as population sizes, Fryxell et al. (2010) suggested that management agencies should reassess the quota levels more often in response to changes in population sizes, and that quota setting should be proportionate to current population size or shifted to threshold approaches.

There is an unavoidable 1‐yr time lag between the availability of monitoring data and the setting harvest quotas for lynx in Scandinavia and a 2‐yr time lag between the availability of monitoring data and the possible evaluation of the effect of harvest quota decision (Fig. 2), which may create some population variability. This extra delay, due to the monitoring being contingent on snow conditions and hunting starting immediately after the monitoring (in Sweden) and before the monitoring season is closed (in Norway), reinforces the need for population models that properly capture all uncertainty in the system. The probability that the forecasted population size will be below the management objectives is considered when the harvest quota is decided, using the model presented here. In effect, this should reduce the risk of undesirable changes in population size. Furthermore, the past decision process for setting the lynx harvest quota in both northern Sweden and Norway suggest that there is a threshold with an increasing proportion above this threshold (Fig. 4). The existence of a threshold was more pronounced in northern Sweden where the overall quota was 0 for the harvest season in March 2015, when the lynx monitoring showed that the lynx population was at the management objective (66 lynx family groups) in February 2014. Above the estimated threshold, the harvest quota increases more than proportionally in both countries. A threshold quota setting with increasing proportional quotas above the threshold reduces population variability compared to constant harvest or strict proportional harvest, especially under uncertainty (Lande et al. 2003, Fryxell et al. 2010, Sæther et al. 2010).

The annual assessment of the monitoring results, the use of forecasting models, and a threshold harvest approach to quota setting will all reduce the risk of lynx population sizes moving outside the desired goals in both Sweden and Norway. The quota setting strategy in Norway, with higher harvest quotas, a lower threshold, and an overall slope closer to a proportional harvest than in northern Sweden, means that the lynx harvest will have a stronger effect on the lynx population in Norway (Fig. 4). But the growth rate (λ) was higher in Norway than in Sweden, which means that the lynx population will recover faster in Norway than in Sweden if there is no harvest. There was a tendency for decreasing quota filling with increasing quota size in Norway, from about 80% quota filling at a quota of 55 lynx to about 70% quota filling at a quota of 140 lynx (Nilsen et al. 2011b), which may be due to an inability of hunters to respond to increased quotas. This will result in the effective harvest being closer to proportional, i.e., a constant proportion of the population is actually harvested every year. The estimated threshold value for Norway also slightly overlapped 0 (95% BCI −0.20 to 33.1, Table 4). A proportional harvest in combination with a 2‐yr time lag in the decision process (Fig. 2) is likely to cause the observed population fluctuations in the Norwegian lynx population (Nilsen et al. 2011b).

Based on monitoring data and long‐term studies of vital rates, we were able to estimate unobserved additional mortality rates. The additional mortality was higher in northern Sweden compared to both southern and northern Norway (Table 2), which resulted in lower survival in northern Sweden compared with both Norwegian areas (Table 3). This in turn, resulted in a lower potential growth rate (λ) in northern Sweden and thus a lower sustainable level of harvest in northern Sweden compared to Norway. There could be several explanations for these differences in survival between Sweden and Norway. The ecological context in northern Sweden and northern Norway are fairly similar for lynx. Semi‐domestic reindeer are the main prey for lynx in both areas (Mattisson et al. 2011). Reproductive rates in lynx are similar in both areas (Nilsen et al. 2012, Walton et al. 2017, see also posterior distribution for recruitment (**r**); Appendix S1: Fig. S3). Thus, there are no clear ecological differences between northern Sweden and northern Norway that could explain the difference in growth rate between the two areas.

The ecological context in southern Norway is slightly different from the northern parts of both Sweden and Norway, as roe deer are the main prey for lynx in southern Norway (Odden et al. 2006, Nilsen et al. 2009). Reproductive rates are higher in southern Norway (Nilsen et al. 2012, see also posterior distribution for recruitment (**r**); Table 2; Appendix S1: Fig. S3), which can partly explain the higher growth rate of lynx in southern Norway (λ median = 1.19). The availability of roe deer as the main prey for lynx may explain the higher reproductive rate in southern Norway (Nilsen et al. 2012). However, there must be some other explanation for the differences in the derived survival (Φ_*i*_ − ρ_*k*_ $\Phi_{i}^{1/2}$; Table 3; Appendix S1: Fig. S4) between Sweden and Norway.

The differences in derived survival could be apparent survival with a net dispersal of lynx from Sweden to Norway. A net dispersal from Sweden to Norway has been shown for wolverines (*Gulo gulo*; Gervasi et al. 2015, 2019), and the effect was measurable 30–40 km into Sweden from the national border. Female lynx, however, have relatively short dispersal distances, with around 50% of young female lynx settling in the neighborhood of their mothers and only 10% dispersing further than 100 km (Samelius et al. 2011). Most of the lynx population in northern Sweden is also found east of the alpine area, i.e., further away from the border between the two countries (Zetterberg and Tovmo 2017 and Fig. 1). Therefore, it is unlikely that the differences in growth rate between northern Sweden and Norway can be explained by net dispersal of females from Sweden to Norway.

Another explanation could be a higher poaching level on lynx in northern Sweden. Andrén et al. (2006) estimated annual poaching rates at 14% in northern Sweden compared to 7% in southern Norway, which could explain the differences in survival and growth rate. There are some differences in management goals between Sweden and Norway that might also explain differences in poaching rates. The national management goal for lynx is more than three times higher in Sweden (221 family groups, 450,000 km^2^) than in Norway (65 lynx family groups, 273,000 km^2^). The regional management goal for the reindeer husbandry area is also three times higher in Sweden (97 family groups, 225,000 km^2^) than in Norway (32 family groups, 140,000 km^2^), although the number of semi‐domestic reindeer is about the same in Sweden (~250,000 reindeer; Hobbs et al. 2012) and Norway (~240,000 reindeer; Tveraa et al. 2014). Interestingly, the legal harvests have been much higher in Norway than in northern Sweden (Fig. 4). Thus, the result may be an indication that higher legal harvest level may reduce poaching. However, the effect of legal harvest on poaching is probably much more complex than that (Treves 2009). Poaching rate is probably also related to the management goals themselves, i.e., if the goals are viewed as unacceptably high poaching will probably continue if there is legal harvest, which may be the case in northern Sweden.

We present a Bayesian forecasting model that assimilates annual monitoring data on lynx abundance together with results of detailed long‐term research on lynx biology, which has provided detailed estimates of key demographic parameters to support an adaptive management of lynx harvest. The model informs decision makers about the policy risks of alternatives for harvest levels, that is, the probability that a given harvest level will cause the population to exceed or fall below objectives on a short‐term perspective (2–4 yr). The approach we illustrate could be adapted to other populations of large carnivores, or indeed any harvested mammal, worldwide.

## Footnotes

1 www.rovbase.se

2 www.rovbase.no
