# Supplementary material for: Harvest models of small populations of a large carnivore using Bayesian forecasting
Source: Ecol Appl. 2020 Jan 28;30(3):e02063. doi: 10.1002/eap.2063 (PMC7187313; doi:10.1002/eap.2063)
Supplement: Supplementary file 1 [file EAP-30-e02063-s001.pdf]

Supporting Information. Henrik Andrén, N. Thompson Hobbs, Malin Aronsson, Henrik Brøseth, Guillaume Chapron, John D.C. Linnell, John Odden, Jens Persson, and Erlend B. Nilsen. 2020. Harvest models of small populations of a large carnivore using Bayesian forecasting. *Ecological Applications.*

APPENDIX S1

Table S1. Management goals (number of lynx family groups during the monitoring period) and associated management goal intervals for counties in the northern carnivore management region in Sweden (Swedish Environmental Protection Agency 2014), management goals for the three carnivore management regions in Sweden and management goals for the eight carnivore regions in Norway (Norwegian Ministry of the Environment 2003).

Management region	Geographical area	Management goal	Management interval
Northern Sweden			
Jämtland (Z)	1	24	20-28
Västernorrland (Y)	1	18	16-24
Västerbotten (AC)	1	23	14-32
Norrbotten (BD)	1	32	18-43
Total Northern Sweden		97	68-127
Total Central Sweden*		84	
Total Southern Sweden*		40	
Total Sweden		221 §	
Norway			
Region 1*		0	
Region 2	2	12	
Region 3	2	5	
Region 4	2	6	
Region 5	2	10	
Region 6	3	12	
Region 7	3	10	
Region 8	3	10	
Total Norway		65	

* Central and southern management regions in Sweden and management region 1 in Norway were not included in this study.
§ This number is the management goal. There is also a minimum level of 147 lynx family groups for Sweden.

Table S2. Sensitivity analyses for demographic parameters with informed priors and initial census result on the potential growth rate (λ). The prior had vague distribution (uniform(0,1)) for one demographic parameter at the time (Table 2). An increase in the standard deviation by 30 % for the number of family groups per total number of females in the population. The initial census result was increased and decreased by 30 % to estimate the effect on potential growth rate (λ). All results were compared with the original population model.

	Definition	Potential population growth rate (λ_1) Northern Sweden Mean ± SD (CV)	Potential population growth rate (λ_2) Southern Norway Mean ± SD (CV)	Potential population growth rate (λ_3) Northern Norway Mean ± SD (CV)
	Original population model (from Table 3)	1.01 ± 0.023 (2.3 %)	1.19 ± 0.030 (2.5 %)	1.16 ± 0.032 (2.8 %)
Φ_1	Probability of survival of subadult females	1.01 ±0.023 (2.3 %)	1.18 ± 0.029 (2.5 %)	1.16 ± 0.30 (2.6 %)
Φ_2	Probability of survival of adult females	1.01 ± 0.022 (2.2 %)	1.19 ± 0.030 (2.5 %)	1.16 ± 0.031 (2.7 %)
r_1	Number of female kittens surviving to census per 2 years old female	1.01 ± 0.021 (2.1 %)	1.18 ± 0.030 (2.5 %)	1.16 ± 0.032 (2.8 %)
r_2	Number of female kittens surviving to census per 3 years and older female	1.01 ± 0.023 (2.3 %)	1.18 ± 0.032 (2.7 %)	1.16 ± 0.034 (2.9 %)
F_k	Number of family groups per total number of females in the population	1.01 ± 0. 023 (2.3 %)	1.17 ± 0.032 (2.7 %)	1.16 ± 0.033 (2.8 %)
$y_{j,k,1}$	Initial census results (30 % higher)	1.00 ± 0.024 (2.4 %)	1.17 ± 0.031 (2.6 %)	1.15 ± 0.033 (2.9 %)
$y_{j,k,1}$	Initial census results (30 % lower)	1.02 ± 0.022 (2.4 %)	1.20 ± 0.031 (2.6 %)	1.17 ± 0.030 (2.6 %)

Table S3. Forecasting number of lynx family groups one year beyond the data. Management objectives, the median number of lynx family groups in 2018 for northern Sweden and Norway with 95% equal-tailed Bayesian credible intervals (BCI), including the known lynx harvest that occurred during 2017. P( ) gives the probability that the future number of lynx family groups will be below, within, or above management objectives for a management region.

Management region	Objective	Median	95 % BCI	P(below)	P(within)	P(above)
Z	20 – 28	29	17 – 48	0.08	0.38	0.54
Y	16 – 24	17	10 – 29	0.43	0.48	0.09
AC	14 – 32	22	13 – 37	0.06	0.87	0.07
BD	18 – 43	24	14 – 40	0.14	0.85	0.01
Total northern Sweden	68 – 127	92	67 – 129	0.03	0.94	0.03
2	12	11	5 – 20	0.66	–	0.34
3	5	6	3 – 12	0.33	–	0.67
4	6	5	2 – 9	0.76	–	0.27
5	10	10	6 – 18	0.48	–	0.52
6	12	13	6 – 24	0.45	–	0.55
7	10	7	4 – 14	0.85	–	0.15
8	10	8	4 – 15	0.73	–	0.27
Total Norway	65	61	46 – 82	0.65	–	0.35

Table S4. Forecasting number of lynx family groups two years beyond the data. Management objectives, the median number of lynx family groups in 2019 for northern Sweden with 95% equal-tailed Bayesian credible intervals (BCI), assuming four different harvest levels during 2018. P( ) gives the probability that the future number of lynx family groups will be below, within, or above management objectives for a management region at the given harvest level.

Management region	Objective	Harvest	Median	95 % BCI	P(below)	P(within)	P(above)
Z	20 – 28	0	30	16 – 56	0.12	0.33	0.56
		5	29	15 – 55	0.14	0.34	0.53
		10	28	14 – 54	0.16	0.35	0.50
		20	27	13 – 52	0.21	0.36	0.44
Y	16 – 24	0	17	9 – 33	0.42	0.43	0.15
		5	16	8 – 32	0.47	0.40	0.13
		10	15	7 – 31	0.52	0.37	0.11
		20	14	6 – 30	0.62	0.30	0.08
AC	14 – 32	0	22	12 – 43	0.08	0.79	0.13
		5	21	11 – 42	0.11	0.78	0.11
		10	20	10 – 41	0.13	0.77	0.10
		20	19	9 – 39	0.19	0.73	0.08
BD	18 – 43	0	25	13 – 47	0.17	0.79	0.04
		5	24	12 – 46	0.20	0.76	0.04
		10	23	12 – 45	0.23	0.74	0.03
		20	22	11 – 43	0.30	0.67	0.03
Total northern Sweden	68 – 127	0	96	65 – 143	0.04	0.88	0.08
		20	93	63 – 139	0.06	0.88	0.06
		40	91	61 – 136	0.08	0.87	0.05
		80	85	56 – 129	0.15	0.82	0.03

Table S5. Forecasting number of lynx family groups two years beyond the data. Management objectives, the median number of lynx family groups in 2019 for Norway with 95% equal-tailed Bayesian credible intervals (BCI), assuming four different harvest levels during 2018. P( ) gives the probability that the future number of lynx family groups will be below or above management objectives for a management region at the given harvest level.

Management region	Objective	Harvest	Median	95 % BCI	P(below)	P(above)
2	12	0	13	6 – 26	0.44	0.56
		5	12	5 – 25	0.51	0.49
		10	11	4 – 24	0.57	0.43
		20	10	3 – 22	0.69	0.31
3	5	0	7	3 – 15	0.20	0.80
		5	6	2 – 14	0.31	0.69
		10	5	2 – 13	0.42	0.58
		20	4	1 – 12	0.64	0.36
4	6	0	6	3 – 12	0.55	0.45
		5	5	2 – 11	0.68	0.32
		10	4	1 – 10	0.78	0.22
		20	3	0 – 8	0.91	0.09
5	10	0	12	6 – 24	0.28	0.72
		5	11	5 – 23	0.35	0.65
		10	10	5 – 22	0.43	0.57
		20	9	4 – 20	0.58	0.42
6	12	0	15	7 – 32	0.30	0.70
		5	14	6 – 31	0.36	0.64
		10	13	5 – 30	0.41	0.59
		20	12	4 – 28	0.53	0.47
7	10	0	8	4 – 18	0.68	0.32
		5	7	3 – 17	0.75	0.25
		10	6	2 – 16	0.81	0.19
		20	5	1 – 14	0.89	0.11
8	10	0	10	5 – 20	0.54	0.46
		5	9	4 – 19	0.62	0.38
		10	8	3 – 18	0.69	0.31
		20	7	2 – 17	0.81	0.19
Total Norway	65	0	74	53 – 104	0.22	0.78
		35	69	48 – 98	0.37	0.63
		70	63	43 – 92	0.55	0.45
		140	53	33 – 80	0.84	0.16

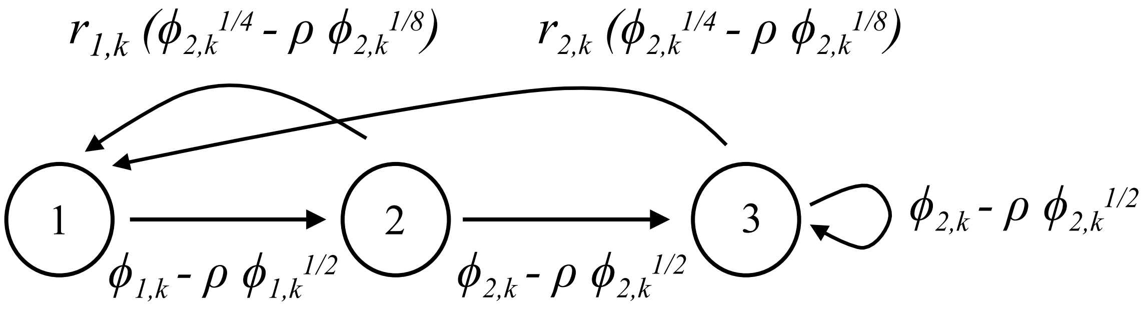

FIG. S1. Life cycle diagram of projection matrix for lynx harvest model. Parameters are ϕ_1 survival of females from 9 to 21 months of age, ϕ_2 survival after 21 months of age, r_1 number of female kittens per 2-years old female at time *t* that survive to census at time *t+1*, r_2 number of female kittens per 3-years old and older female at time *t* that survive to census at time *t+1*, ρ additional mortality and *k* geographical area.

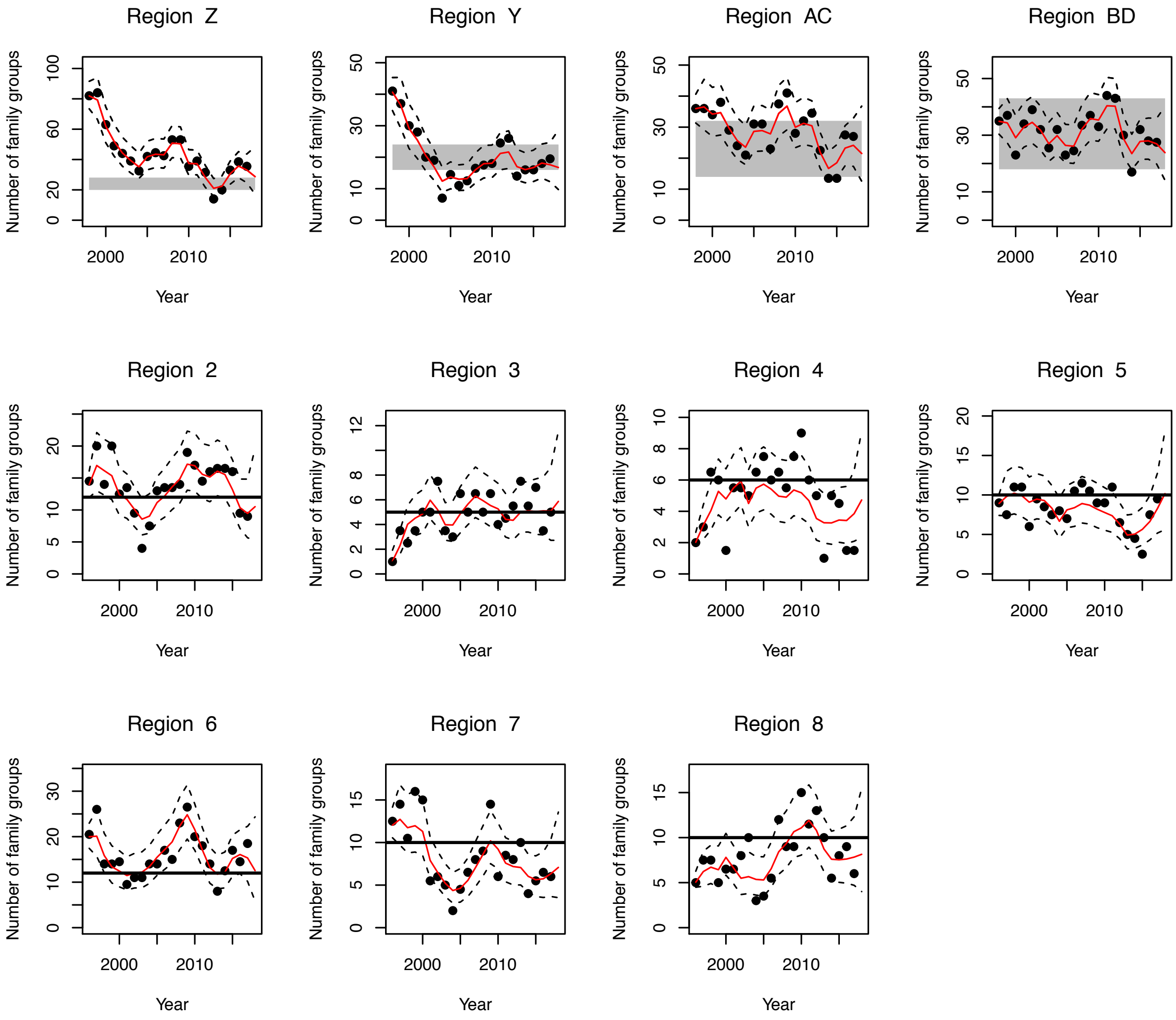

FIG. S2. Medians of posterior distributions of the estimated number of lynx family groups in northern Sweden and Norway (solid red line) and 95% equal-tailed Bayesian credible intervals (dashed lines). Black dots show the monitoring data. A model forecast extends one year beyond the data. Grey shaded bar shows the acceptable upper and lower limits for the number of lynx family groups in northern Sweden (Management regions Z, Y, AC and BD) and the black line shows the objectives for number of lynx family groups in Norway (Management regions 2-8).

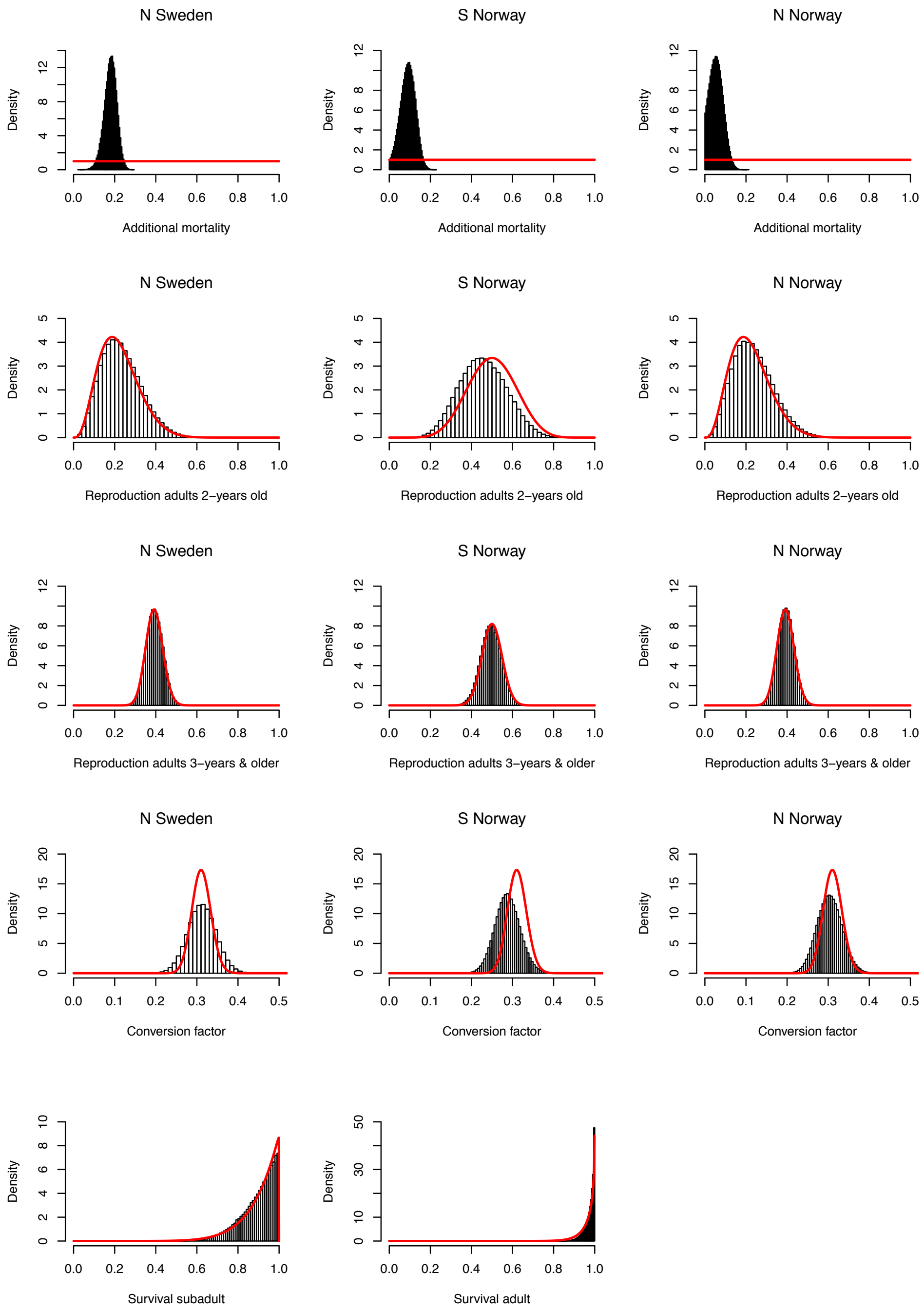

FIG. S3. Prior distributions of parameters (red lines) and their posterior distributions (histograms) of the coefficients in the lynx harvest model. Parameters are geographical specific ρ additional mortality, r_1 number of female kittens surviving to census per 2-years old female and r_2 number of female kittens surviving to census per 3-years old and older female and F (conversion factor) the number of family groups per total number of females in a lynx population in northern Sweden, southern Norway and northern Norway and ϕ_1 survival of females from 9 to 21 months of age, ϕ_2 survival after 21 months of age independent of geographical area.

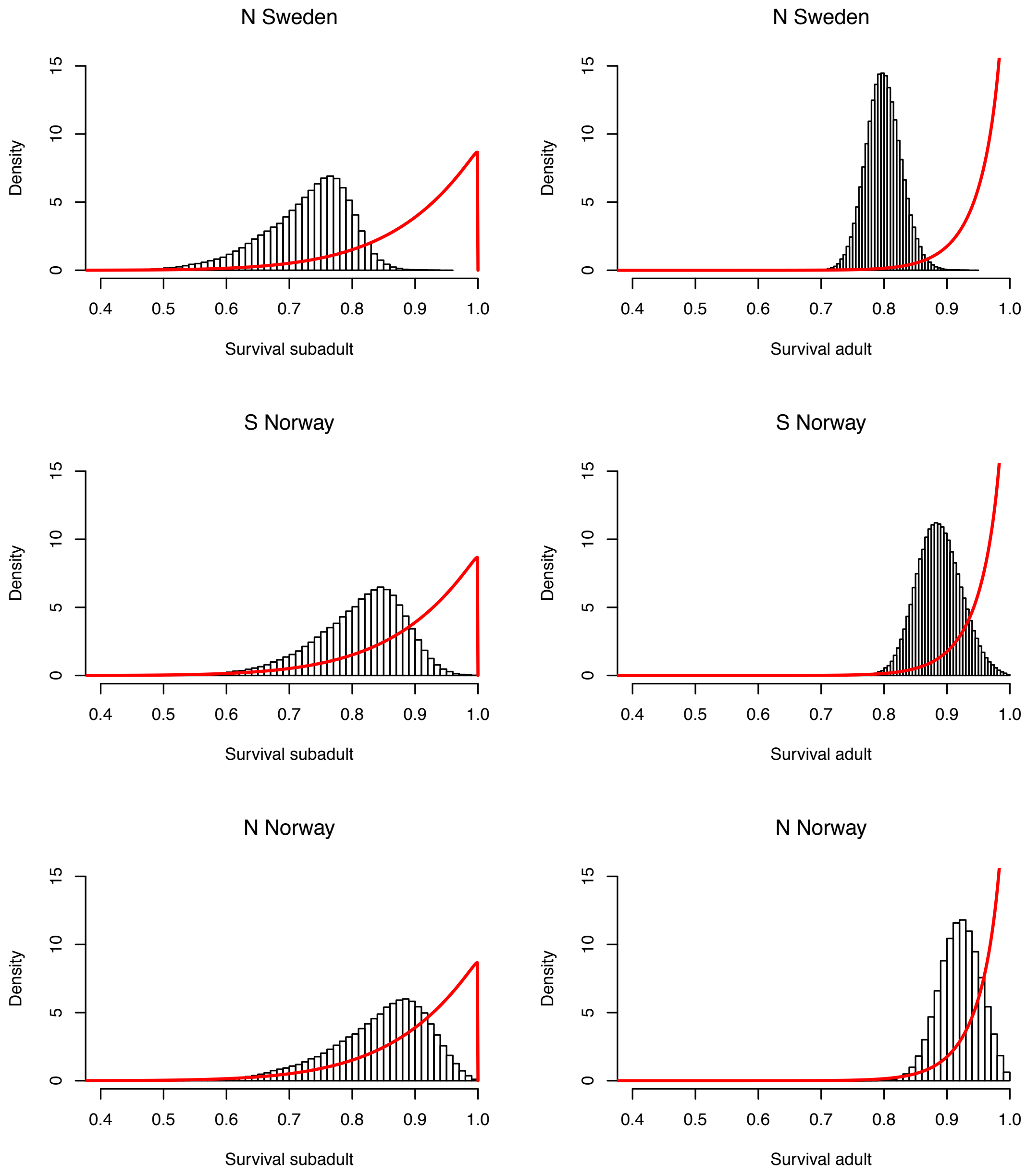

FIG. S4. Posterior distributions of the derived survival estimates ($\Phi_{i,k}$ - $\rho_k\Phi_{i,k}{}^{1/2}$) in the lynx model and the prior survival distribution (red line, from Table 2) for subadult and adult females in the three geographic areas (northern Sweden, southern Norway and northern Norway).

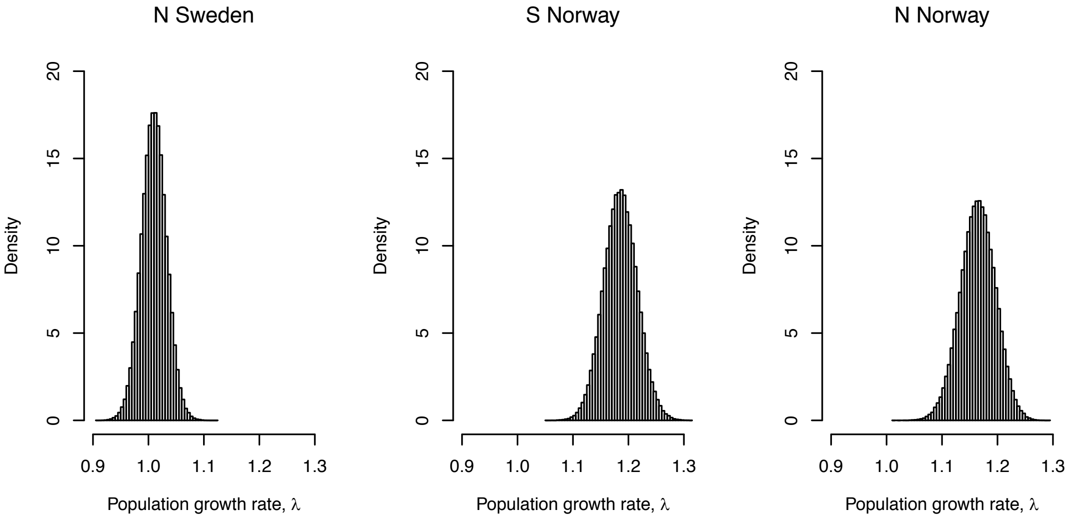

FIG. S5. Posterior distributions of potential lynx population growth rates in the three geographic areas (northern Sweden, southern Norway and northern Norway).

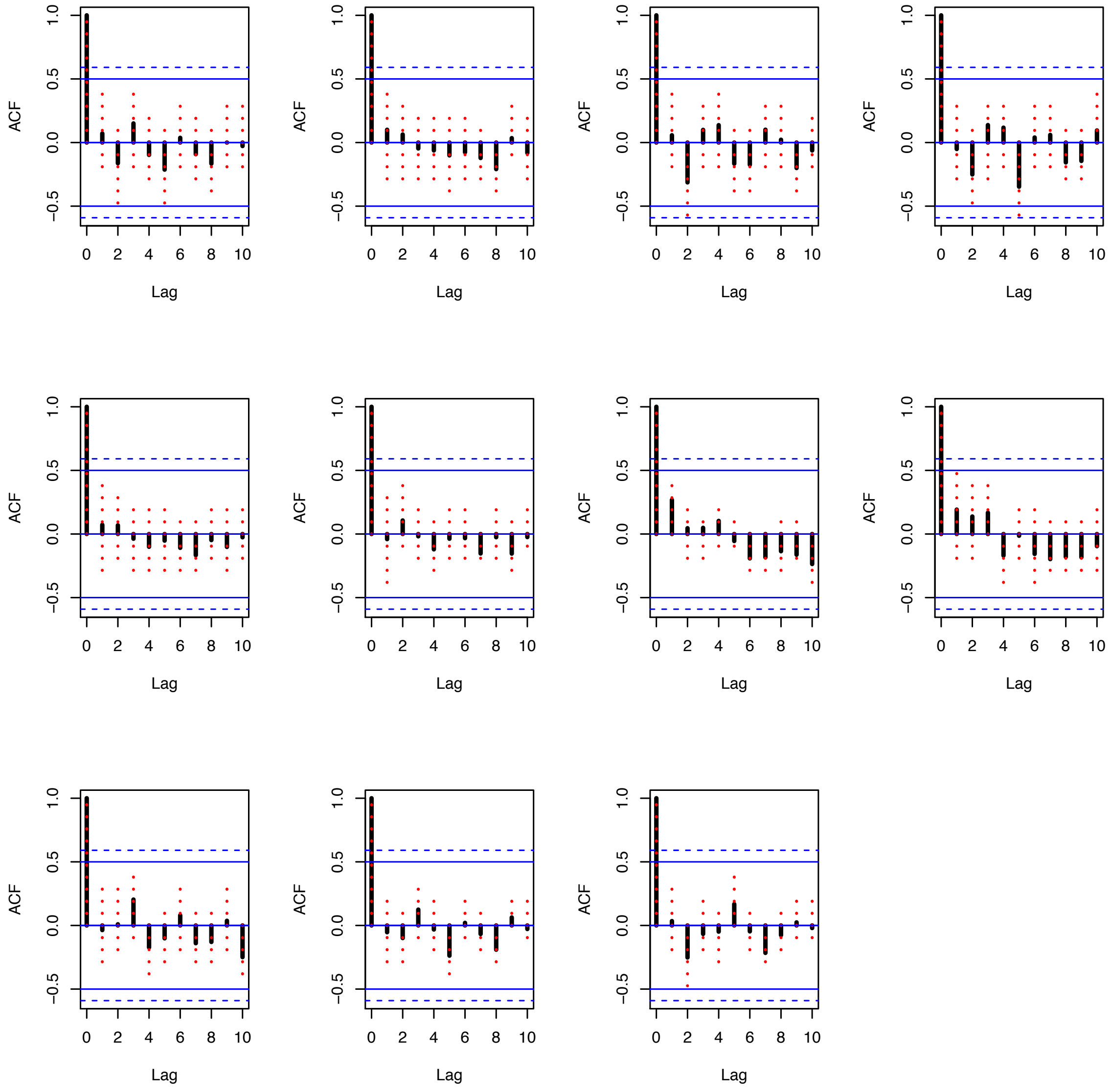

FIG. S6. Plot of autocorrelation functions for 11 management regions in northern Sweden and Norway used to model lynx population dynamics. Dark, solid bars lines indicate the ACF (auto correlation function) scores on the function at lags from 0 to 10 years. Failure of these bars to cross the blue, dotted horizontal lines allows rejection of the hypothesis of significant autocorrelation. Dashed, red lines give 95% confidence limit of the ACF score. Solid blue lines indicate 0.50 correlations.

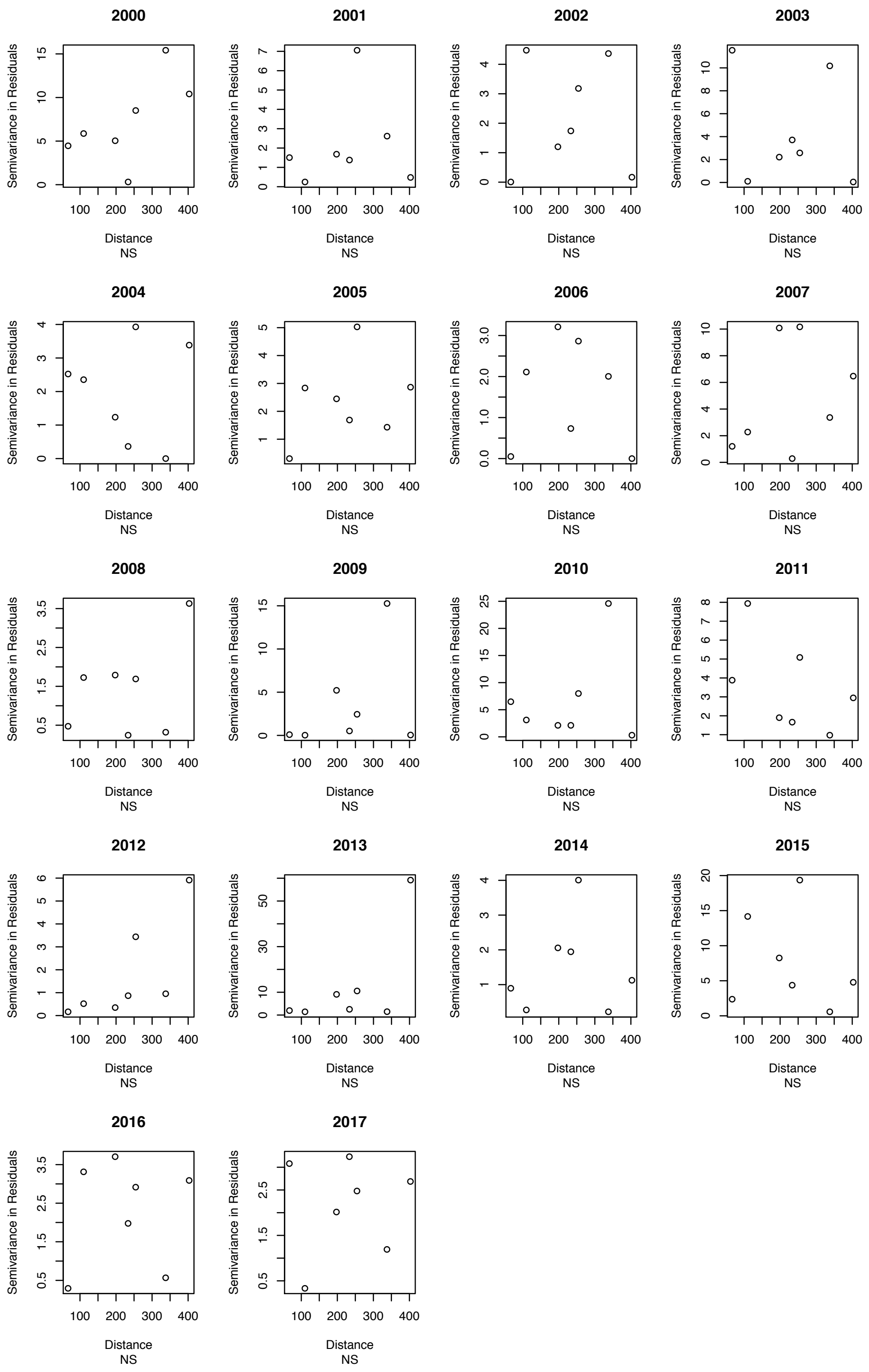

FIG. S7. Semi-variograms of residuals in north-south direction for 11 management regions in northern Sweden and Norway. There was no north-south spatial dependence, as the residuals were not correlated with distance in any of the years.

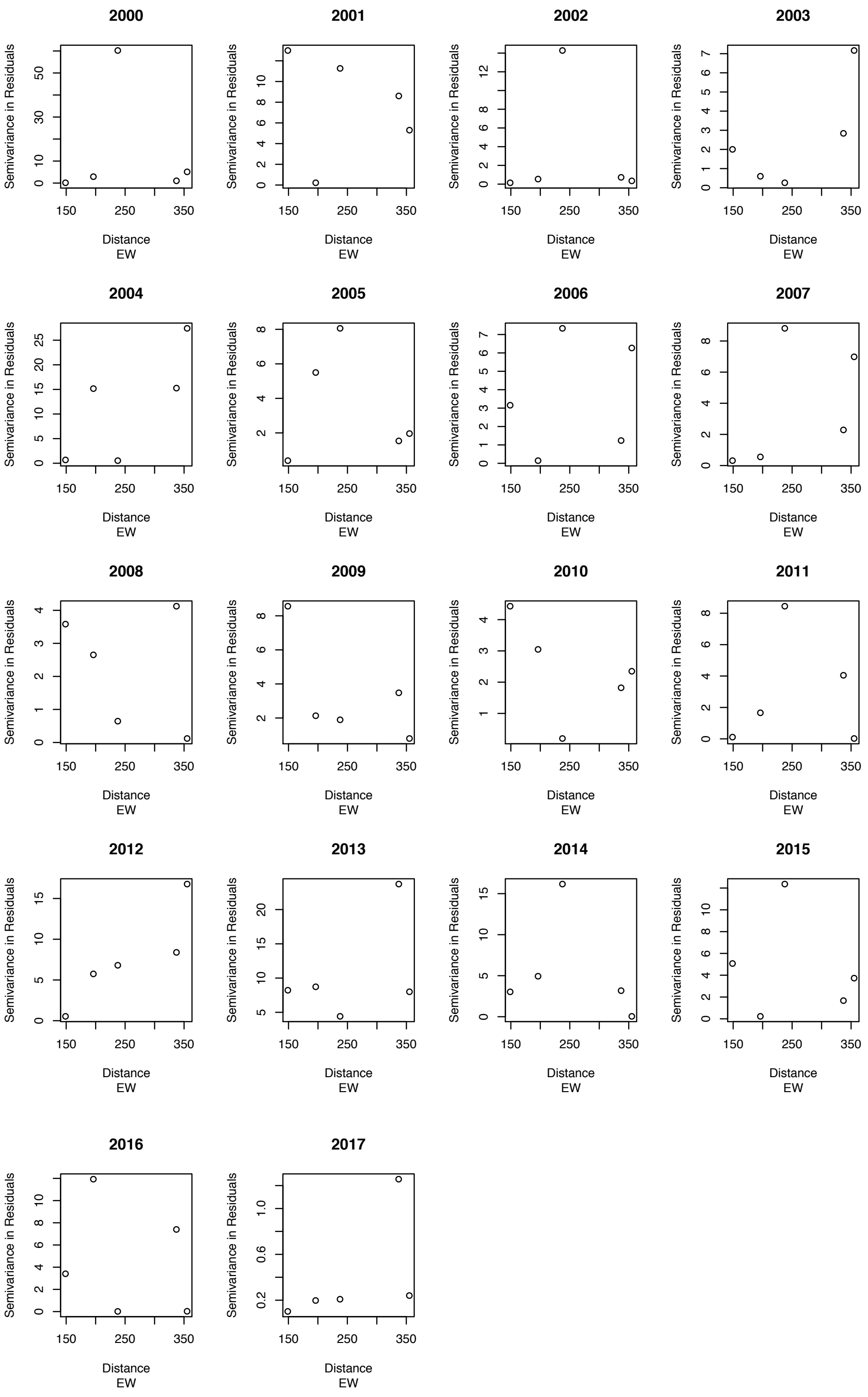

FIG. S8. Semi variograms of residuals in east-west direction for 11 management regions in northern Sweden and Norway. There was no east-west spatial dependence, as the residuals were not correlated with distance in any of the years.

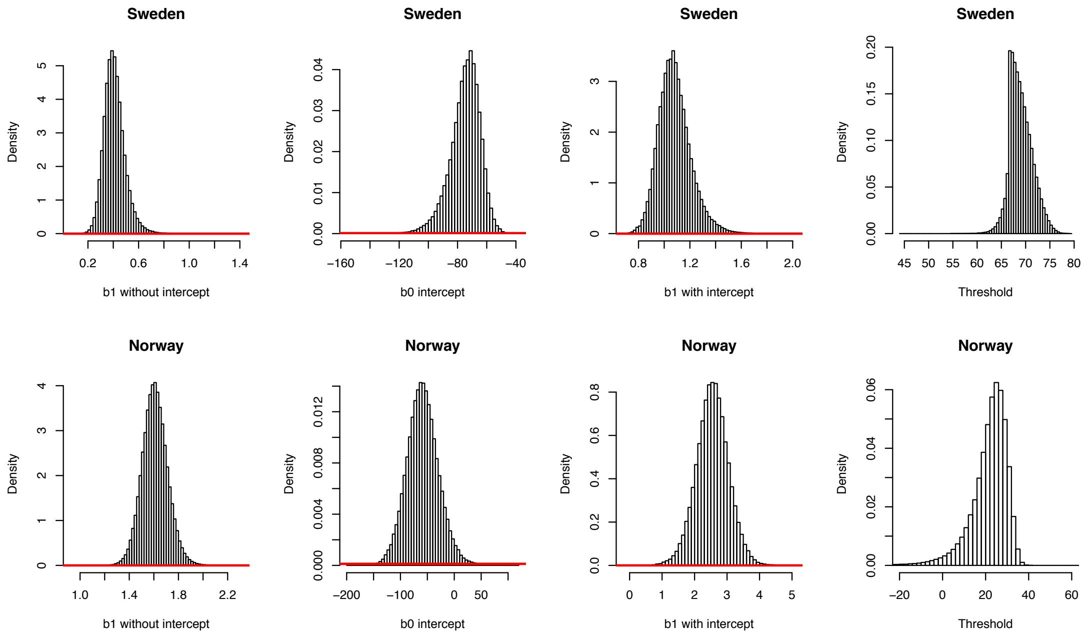

FIG. S9. Prior distributions of parameters (red lines) and their posterior distributions (histograms) of the coefficients in the lynx quota decision models and posterior distributions of the derived threshold ($-b_0/b_1$) in number of lynx family groups below which there will be no harvest based past lynx quota decisions.
