# Supplementary material for: Harvest models of small populations of a large carnivore using Bayesian forecasting
Source: Ecol Appl. 2020 Jan 28;30(3):e02063. doi: 10.1002/eap.2063 (PMC7187313; doi:10.1002/eap.2063)
Supplement: Supplementary file 2 [file EAP-30-e02063-s002.pdf]

**Henrik Andrén, N. Thompson Hobbs, Malin Aronsson, Henrik Brøseth, Guillaume Chapron, John D.C. Linnell, John Odden, Jens Persson, and Erlend B. Nilsen. 2020. Harvest models of small populations of a large carnivore using Bayesian forecasting. *Ecological Applications.***

---

**Data S1**

Andren Data Harvest model.csv
Andren Data Quota model.csv

---

**Authors**

Henrik Andrén, Grimsö Wildlife Research Station, Department of Ecology, Swedish University of Agricultural Sciences, SE-730 91 Riddarhyttan, Sweden, e-mail: henrik.andren@slu.se

N. Thompson Hobbs, Natural Resource Ecology Laboratory, Department of Ecosystem Science and Sustainability, and Graduate Degree Program in Ecology, Colorado State University, Fort Collins, Colorado 80523, USA

Malin Aronsson, Grimsö Wildlife Research Station, Department of Ecology, Swedish University of Agricultural Sciences, SE-730 91 Riddarhyttan, Sweden, e-mail: malin.aronsson@slu.se

Henrik Brøseth, Rovdata, Norwegian Institute for Nature Research, P.O. Box 5685, Torgard, NO-7485 Trondheim, Norway, e-mail: henrik.Broseth@nina.no

Guillaume Chapron, Grimsö Wildlife Research Station, Department of Ecology, Swedish University of Agricultural Sciences, SE-730 91 Riddarhyttan, Sweden, e-mail: guillaume.chapron@slu.se

John D.C. Linnell, Norwegian Institute for Nature Research, P.O. Box 5685, Torgard, NO-7485 Trondheim, Norway, e-mail: john.linnell@nina.no

John Odden, Norwegian Institute for Nature Research, P.O. Box 5685, Torgard, NO-7485 Trondheim, Norway, e-mail: john.odden@nina.no

Jens Persson, Grimsö Wildlife Research Station, Department of Ecology, Swedish University of Agricultural Sciences, SE-730 91 Riddarhyttan, Sweden, e-mail: jens.persson@slu.se

Erlend B. Nilsen, Norwegian Institute for Nature Research, P.O. Box 5685, Torgard, NO-7485 Trondheim, Norway, e-mail: erlend.nilsen@nina.no

**File list (files found within DataS1.zip)**

```
Andren Data Harvest model.csv
Andren Data Quota model.csv
```

**Description**

`Andren Data Harvest model.csv` contains the monitoring and harvest data for the lynx harvest model.

The columns are:

`year` – the year of census (February)

`run` – the run in the data

`country` – code for country; S = Sweden and N = Norway

`region` – code for management region; Z = Jämtland, Y = Västernorrland, AC = Västerbotten, BD = Norrbotten, 2 – 8 = the different large carnivore management regions in Norway (2 – 8)

`census` – number of lynx family groups censused in that year in that region

`harvest` – total number of lynx harvested in that year in that region

`harvest_F_>1yr` – number of females older than one year harvested in that year in that region

`harvest_F_kitten` – number of female kittens (10 months old) harvested in that year in that region

`Andren Data Quota model.csv` contains the monitoring and quota data for the lynx quota decision model.

The columns are:

`year` – the year of census (February)

`run` – the run in the data

`country` – code for country; 1 = Sweden and 2 = Norway

`census` – number of lynx family groups censused in that year in that region

`quota` – the harvest quota for lynx based on the census result of the same year in that region

`quota_1` – the harvest quota for lynx based on the census result of the year before in the region
